# Effect of fracture risk in inhaled corticosteroids in patients with chronic obstructive pulmonary disease: a systematic review and meta-analysis

**DOI:** 10.1186/s12890-023-02602-5

**Published:** 2023-08-17

**Authors:** Shisheng Peng, Cong Tan, Lirong Du, Yanan Niu, Xiansheng Liu, Ruiying Wang

**Affiliations:** 1 Third Hospital of Shanxi Medical University, Shanxi Bethune Hospital, Shanxi Academy of Medical Sciences, Tongji Shanxi Hospital, Taiyuan, 030032 China; 2 Department of Respiratory and Critical Care Medicine, National Clinical Research Center of Respiratory Disease, Tongji Hospital, Tongji Medical College, Huazhong University of Science and Technology, Wuhan, 430030 China

**Keywords:** Inhaled glucocorticoids, Fracture, COPD, Triple therapy

## Abstract

**Background:**

The fracture risk of patients with chronic obstructive pulmonary disease (COPD) treated with inhaled corticosteroids is controversial. And some large-scale randomized controlled trials have not solved this problem. The purpose of our systematic review and meta-analysis including 44 RCTs is to reveal the effect of inhaled corticosteroids on the fracture risk of COPD patients.

**Methods:**

Two reviewers independently retrieved randomized controlled trials of inhaled corticosteroids or combinations of inhaled corticosteroids in the treatment of COPD from PubMed, Embase, Medline, Cochrane Library, and Web of Science. The primary outcome was a fracture event. This study was registered at PROSPERO (CRD42022366778).

**Results:**

Forty-four RCTs were performed in 87,594 patients. Inhaled therapy containing ICSs (RR, 1.19; 95%CI, 1.04–1.37; *P* = 0.010), especially ICS/LABA (RR, 1.30; 95%CI, 1.10–1.53; *P* = 0.002) and triple therapy (RR, 1.49; 95%CI, 1.03–2.17; *P* = 0.04) were significantly associated with the increased risk of fracture in COPD patients when compared with inhaled therapy without ICSs. Subgroup analyses showed that treatment duration ≥ 12 months (RR, 1.19; 95%CI, 1.04–1.38; *P* = 0.01), budesonide therapy (RR, 1.64; 95%CI., 1.07–2.51; *P* = 0.02), fluticasone furoate therapy (RR, 1.37; 95%CI, 1.05–1.78; *P* = 0.02), mean age of study participants ≥ 65 (RR, 1.27; 95%CI, 1.01–1.61; *P* = 0.04), and GOLD stage III(RR, 1.18; 95%CI, 1.00–1.38; *P* = 0.04) were significantly associated with an increased risk of fracture. In addition, budesonide ≥ 320 ug bid via MDI (RR, 1.75; 95%CI, 1.07–2.87; *P* = 0.03) was significantly associated with the increased risk of fracture.

**Conclusion:**

Inhalation therapy with ICSs, especially ICS/LABA or triple therapy, increased the risk of fracture in patients with COPD compared with inhaled therapy without ICS. Treatment duration, mean age of participants, GOLD stage, drug dosage form, and drug dose participated in this association. Moreover, different inhalation devices of the same drug also had differences in risk of fracture.

**Supplementary Information:**

The online version contains supplementary material available at 10.1186/s12890-023-02602-5.

Chronic obstructive pulmonary disease (COPD) is a heterogeneous airway disease, characterized by persistent respiratory symptoms and gradual airflow limitation [1]. With high morbidity and mortality, COPD is the third leading cause of death in the world [2]. Repeated acute exacerbations of COPD patients increase the frequency of hospitalization and are related to poor prognosis [3]. Inhaled corticosteroids (ICSs) and long-acting β_2_-agonists (LABAs) and long-acting muscarinic receptor antagonists (LAMAs) are three independent inhaled drugs, which can be used alone or in combination in the process of the disease progression to reduce the burden of COPD [1]. But, there is still much debate on the appropriate prescription of ICS in patients with COPD. The Global Initiative for Chronic Obstructive Pulmonary Disease (GOLD) suggests ICS use has been restricted only to selected COPD patients mainly based on the risk of exacerbations, high blood eosinophilia, or asthmatic [1]. However, some discrepancies between treatment recommendations and real-life use of ICS were found in surveys performed in many countries [4–6]. The current situation of COPD treatment in different regions showed that more than 50% of newly diagnosed patients with COPD receiving ICS-based treatment from the start [5, 6]. Hence, evidences and guidelines are becoming increasingly clear about the imbalance between the risks and benefits of ICSs in patients with COPD.

Although ICS can reduce the risk of exacerbation in COPD patients, it is reported that ICS increase the risk of adverse events, such as pneumonia [7] and upper respiratory infection [8]. It has also been reported that ICSs may increase the risk of fracture events in patients with COPD [9, 10]. In particular, most COPD patients are elderly and have various complications, and with the increase in age long-term inhalation of glucocorticoid may aggravate this risk [11]. Some large randomized controlled trials (RCTs) reported the fracture events of COPD patients treated with ICS, but most of these studies failed to determine the significant difference in fracture risk between ICS treatment group and non-ICS group [12, 13]. According to the TORCH (6112 patients) study in 2007, there is no difference in fracture risk in patients with COPD treated with inhalation therapy containing fluticasone propionate compared with salmeterol and placebo [12]. The adverse event analysis of the SUMMIT trial (23,835 patients) in 2016 showed that there was no difference in fracture risk between ICS treatment group and non-ICS treatment group [13].

Currently, it is still controversial that inhaled corticosteroids increase the risk of fracture in patients with COPD. Whether inhaled glucocorticoids increase the risk of fracture in patients with COPD may depend on the timing, dose, and dosage form of the ICSs treatment. Therefore, we performed a meta-analysis of randomized controlled trials to assess the relationship between ICSs use and fracture risk in patients with COPD. We also aimed to assess the contribution of ICS/LABA and triple therapy on fracture risks.

## Methods

This study was conducted according to the Preferred Reporting Item statement for system review and meta-analysis (PRISMA) [14] and was registered with PROSPERO (CRD42022366778).

### Search strategy

Two reviewers independently retrieved articles from PubMed, Embase, Medline, Cochrane Library, and Web of Science, starting in October 2022 and updating in November 2022. The text terms related to COPD and ICSs were used. RCTs published in English were included. Details of the study search terms and the specific process are shown in Table S1.

### Selection criteria

Eligible studies were identified by PICOS criteria (participants, interventions, comparators, results and study design) [14]. Inclusion criteria include: (1) patients with COPD; (2) Interventions include any type of inhaled glucocorticoids, including ICSs alone or in combination with LABA and/or LAMA; (3) Non-ICSs treatments are used as the control, including placebo or other drugs that do not contain inhaled corticosteroids; (4) Trials that report fracture event data as a result, or trials that report fracture events on ClinicalTrials.GOV; (5) Only randomized controlled trials were included. Exclusion criteria included: (1) non-randomized controlled trials such as observational studies, case series, and reviews; (2) Non-English manuscripts; (3) Patients with asthma or unknown diagnosis; (4) ICS was adopted in both treatment and control groups.

### Data extraction

Two reviewers independently extracted relevant data from included RCTs into standardized collection forms for results and evidence. Differences between the two investigators were resolved through discussions and a third investigator was consulted, as necessary. For articles that did not report all adverse events, we used the information published on ClinicalTrials.gov.

### Risk of bias assessment and quality of evidence

Two reviewers independently performed the risk assessment using Cochrane Collaboration's bias risk tool [15]. The evaluation was performed according to the following characteristics: (1) random sequence generation; (2) distribution concealment; (3) blinding of participant and personnel; (4) Blind method of result evaluation; (5) selective reporting; (6) incomplete of result data; (7) Other biases. Each item was assessed as low, unclear, or high risk of bias. Any differences between the two investigators were resolved through discussions and a third was consulted, as necessary.

### Statistical analysis

We used Revman software (version 5.4, Cochran Collaborative Company, London, UK) and Stata software (version 17.0) to conduct meta-analysis on quantitative meta synthesis. The weight of each study was estimated by Mantel-Hanszel method. We calculated the risk ratio (RR) and 95% confidence interval (CI) of fracture risk. *P* < 0.05 is statistically significant. The heterogeneity was tested by I^2^ test, with I^2^ > 50%, indicating that there was significant heterogeneity. When a large amount of heterogeneity is found, the random effect model will be used; otherwise, the fixed effect model will be used. Publication bias was qualitatively evaluated by a visual funnel diagram, and quantitatively evaluated by the Egger test and the Begg test. We conduct sensitivity analysis by excluding tests that may have the risk of bias. If a *p*-value was less than 0.05 (both tails), the difference was considered statistically significant. We also used the GRADE approach to evaluate the quality of the evidence (Table S3).

### Subgroup analyses

We performed several subgroup analyses based on lengths of follow-up (≥ 12 months and < 12 months); the mean age of study participants (≥ 65 and < 65 years); the severity of COPD (GOLD stage II and GOLD stage III); and whether ICS combined with LAMA or LABA (triple therapy versus LAMA/LABA, triple therapy versus control, ICS/LABA versus LABA, ICS/LABA versus LAMA, ICS/LABA versus LAMA/LABA).

## Results

### Eligible trials

**Fig. 1:**
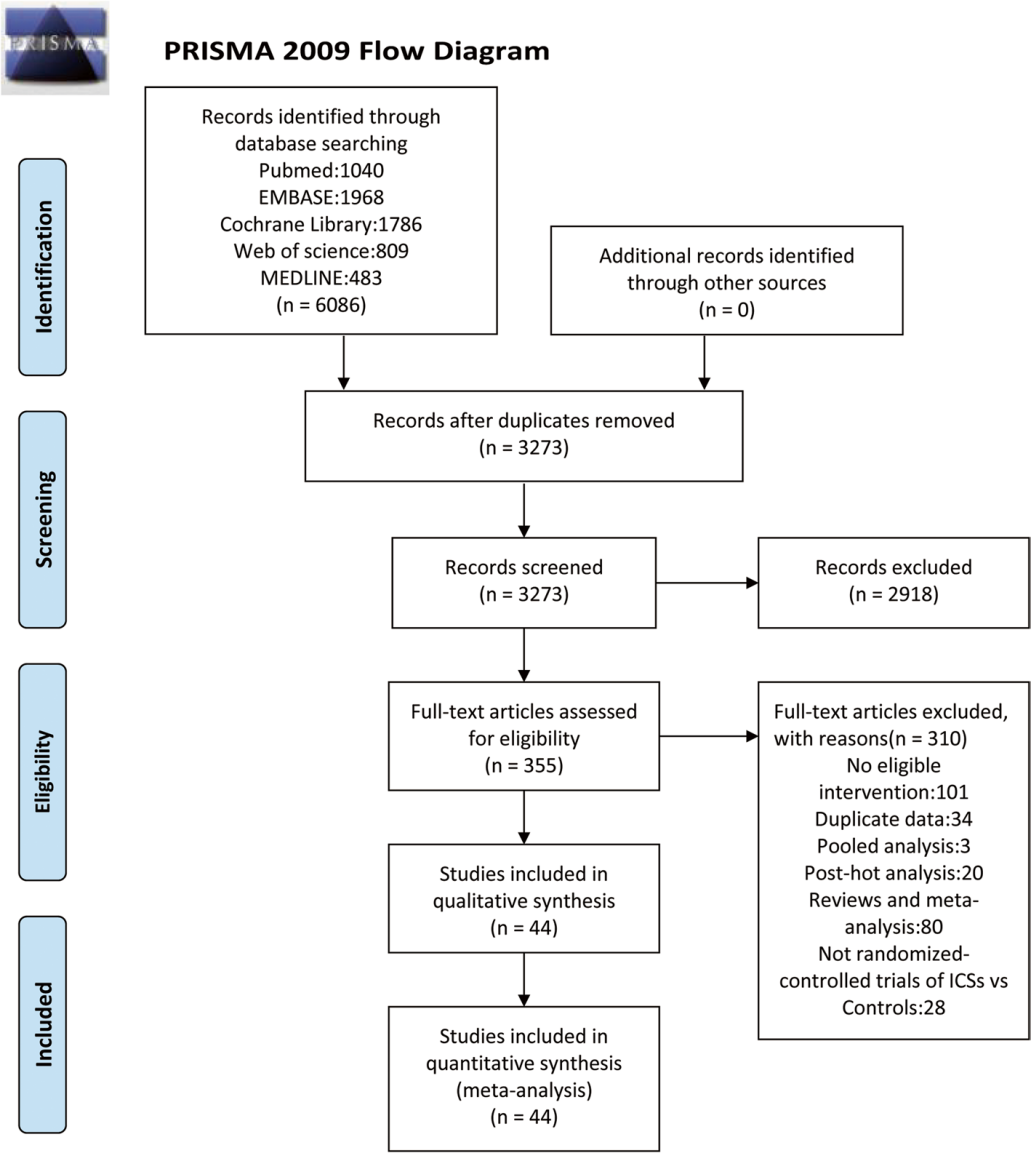
Flow of study selection

A total of 44 eligible RCTs reporting information on fracture were included in the meta-analysis (Fig. 1). The characteristics of the 44 RCT were summarized in Table S2. These 44 RCTs recruited 87,594 subjects in total [12, 13, 16–57]. Of these, 31 RCTs (*N* = 56,250) evaluated ICS/LABA therapy vs. Controls (LAMA only, LABA only, LAMA/LABA, or placebo), and 13 RCTs (*N* = 24,887) assessed ICS/LAMA/LABA vs. Controls (LAMA only, LABA only, LAMA/LABA, or placebo). 7 RCTs had a followed-up of 3 months, 14 had a followed-up of 6 months, 16 had a followed-up of 12 months, 1 had a followed-up of 24 months, and 6 had a followed-up of 36 months.

### Risk of bias

The results of the bias assessment are summarized in Figure S1. Two RCTs were deemed to be at high risk for performance bias. One trial was deemed to be at high risk for detection bias. Two trials were deemed to be at high risk for attrition bias. One trial was deemed to be at high risk for selection bias. Fourteen RCTs were deemed to be at low risk for bias. Information on withdrawal rates was available for all included studies. The approximate symmetry in the funnel plot indicates the absence of substantial publication bias (Figure S2). The results from the Egger test and Begg test also confirmed no published bias (Figure S3).

### Risk of fractures with ICSs therapy vs. Controls

**Fig. 2:**
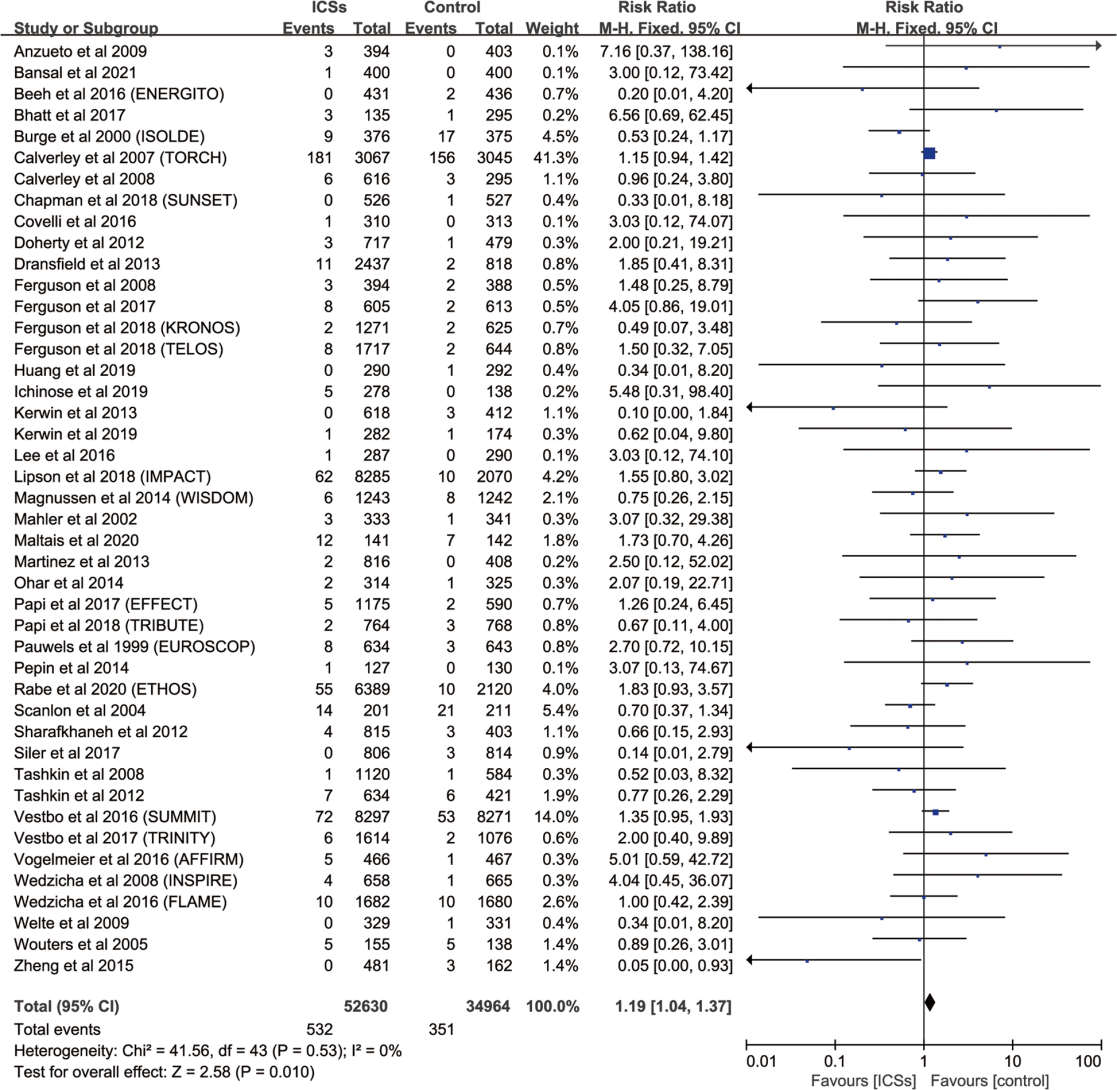
Meta-analysis of included RCTs of ICSs therapy vs. Inhaled therapy without ICSs for fracture risk

Forty-four RCTs enrolling 87,594 patients with COPD were analyzed. Compared with inhaled therapy without ICSs, inhaled therapy containing ICSs was associated significantly with a increased in fractures risk (RR, 1.19; 95%, 1.04–1.37; *P* = 0.010; heterogeneity: *I* ^2^ = 0) (Fig. 2, Table 1).

**Table 1:** Subgroup analysis and GRADE evidence

**Results**	**No. of** **Participants**	**No. of** **Studies**	**Inhaled Therapy** **Containing ICSs**		**Inhaled Therapy** **Without ICSs**		**Risk Ratio** **(M-H, Fixed,95% CI)**	***P*** **value**	**I 2 (%)**	**GRADE** **evidence**
			Events	Patients	Events	Patients				
**Risk of fracture for ICSs vs. controls**	87,594	44	532	52,630	351	34,964	1.19 (1.04,1.37)	**0.010**	0	Moderate
**Risk of fracture for ICSs vs. Controls according to different duration of treatment**										
< 12 months	19,840	21	48	11,486	29	8354	1.20 (0.79,1.81)	0.39	13	Low
≥ 12 months	67,387	23	489	40,970	326	26,417	1.19 (1.04,1.38)	**0.01**	0	High
**Risk of fracture for ICSs vs. Controls according to different type of treatment**										
Triamcinolone	412	1	14	201	21	211	0.70 (0.37,1.34)	0.28	NA	Very low
Mometasone furoate	3162	3	16	1967	10	1195	0.95 (0.43,2.10)	0.9	0	Low
Beclometasone dipropionate	4222	2	8	2378	5	1844	1.26 (0.40,3.99)	0.69	0	Moderate
Budesonide	20,874	12	93	14,017	26	6857	1.64 (1.07,2.51)	**0.02**	0	High
160 ug bid	8256	6	20	4024	17	4232	1.17 (0.62,2.18)	0.63	0	Low
320 ug bid	14,342	9	60	8747	22	5595	166 (1.03,2.70)	**0.04**	0	High
Fluticasone furoate	38,021	13	170	23,319	95	14,702	1.37 (1.05,1.78)	**0.02**	20	Moderate
50 ug qd	2578	3	4	1186	8	1392	0.66 (0.21,2.05)	0.47	32	Moderate
100 ug qd	34,522	11	160	20,287	82	14,235	1.37 (1.04,1.80)	**0.02**	0	Moderate
200 ug qd	2767	3	6	1379	5	1388	1.18 (0.40,3.50)	0.77	37	Moderate
Fluticasone propionate	20,903	13	231	10,748	206	10,155	1.10 (0.92,1.32)	0.62	0	Moderate
250 ug bid	4044	5	11	1902	7	2142	1.72 (0.68,4.34)	0.25	0	Low
500 ug bid	18,898	11	225	9346	204	9552	1.10 (0.92,1.32)	0.31	0	Low
**Risk of fracture for ICSs vs. Controls according to different mean age**										
< 65	45,519	30	183	28,000	115	17,519	1.08 (0.85,1.37)	0.53	3	Low
≥ 65	42,075	14	349	24,630	236	17,445	1.26 (1.07,1.48)	**0.007**	0	Moderate
**Risk of fracture for ICSs vs. Controls according to different GOLD grade**										
GOLD 2	28,500	12	125	15,025	96	13,475	1.26 (0.97,1.63)	0.08	22	Low
GOLD 3	56,585	28	394	36,121	247	20,464	1.18 (1.00,1.38)	**0.04**	0	High
**Risk of fracture for different ICSs vs. controls**										
Triple therapy vs controls	24,887	13	98	14,834	39	10,053	1.49 (1.03,2.17)	**0.04**	0	High
ICS/LABA VS controls	56,250	31	274	28,144	292	28,106	1.30 (1.10,1.53)	**0.002**	0	High
ICSs vs. PLACEBO	17,557	12	164	9037	149	8520	1.07 (0.86,1.33)	0.52	4	Moderate
**Risk of fracture for triple therapy vs. different controls**										
triple therapy vs LAMA/LABA	19,578	8	90	11,914	35	7664	1.51 (1.01,2.25)	**0.04**	0	High
triple therapy vs LAMA	5309	5	8	2920	4	2389	1.38 (0.49,3.88)	0.54	0	Low
**Risk of fracture for triple therapy vs. LAMA/LABA according to different duration of treatment**										
6 months	2594	3	2	1304	3	1290	0.74 (0.17,3.31)	0.7	0	Low
12 months	16,984	5	88	10,610	32	6374	1.59 (1.05,2.41)	**0.03**	0	High
**Risk of fracture for triple therapy vs. LAMA/LABA according to different mean age**										
< 65	10,763	4	45	6459	22	4304	1.29 (0.76,2.20)	0.34	0	Moderate
≥ 65	8815	4	45	5455	13	3360	1.82 (0.99,3.36)	0.06	0	Low
**Risk of fracture for triple therapy vs. LAMA/LABA according to different GOLD grade**										
GOLD 2	2317	2	1	1165	3	1152	0.42 (0.06,2.86)	0.38	0	Moderate
GOLD 3	16,616	4	87	10,416	31	6200	1.61 (1.05,2.45)	**0.03**	19	High
**Risk of fracture for ICS/LABA vs. different controls**										
ICS/LABA VS LAMA/LABA	17,413	8	63	9703	36	7710	1.28 (0.94,2.10)	0.10	0	Moderate
ICS/LABA VS LABA	29,059	19	204	16,865	146	12,194	1.24 (1.01,1.44)	**0.04**	0	High
ICS/LABA VS LAMA	2203	3	6	1095	1	1108	3.55 (0.74,17.03)	0.11	0	Moderate
ICS/LABA VS PLACEBO	13,249	6	143	6878	105	6371	1.32 (1.04,1.69)	**0.02**	35	Moderate
**Risk of fracture for ICS/LABA vs. LABA according to different duration of treatment**										
3 months	1620	1	0	806	3	814	0.14 (0.01, 2.79)	0.2	NA	Low
6 months	6372	7	22	3987	9	2385	1.67 (0.83, 3.37)	0.15	7	Moderate
12 months	9450	8	36	6258	19	3192	1.07 (0.62, 1.83)	0.82	0	Low
36 months	11,617	3	146	5814	115	5803	1.26 (1.00, 1.47)	0.05	0	High
**Risk of fracture for ICS/LABA vs. LABA according to different mean age**										
< 65	14,736	13	52	9716	27	5020	1.12 (0.72, 1.76)	0.61	0	Moderate
≥ 65	14,323	6	152	7149	119	7174	1.27 (1.01, 1.61)	**0.04**	0	Moderate
**Risk of fracture for ICS/LABA vs. LABA according to different GOLD grade**										
GOLD 2	12,627	5	59	6733	43	5894	1.29 (0.87,1.90)	0.20	0	Moderate
GOLD 3	15,799	13	141	9708	98	6091	1.27 (0.99,1.63)	0.06	0	High

Subgroup analysis based on duration of follow-up revealed that ICSs (23 RCTs; RR, 1.19; 95%CI, 1.04–1.38; *P* = 0.01; heterogeneity: *I* ^2^ = 0) was associated with a significantly increased the risk of fractures compared with control in patients who continue the treatment for at least 12 months (Figure S8).

**Fig. 3:**
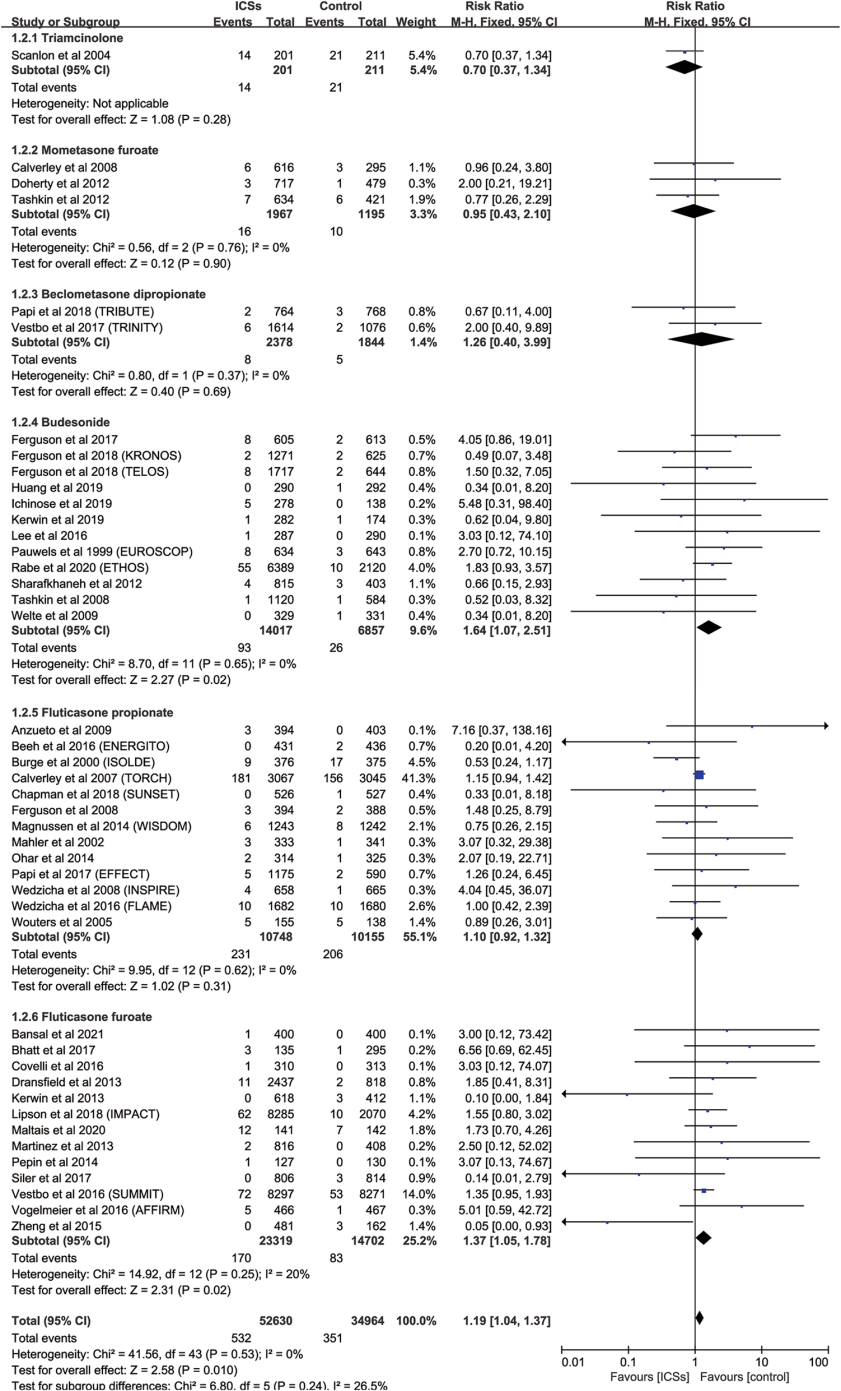
Risk of fractures with ICSs therapy vs. Inhaled therapy without ICSs according to different dosage forms

**Fig. 4:**
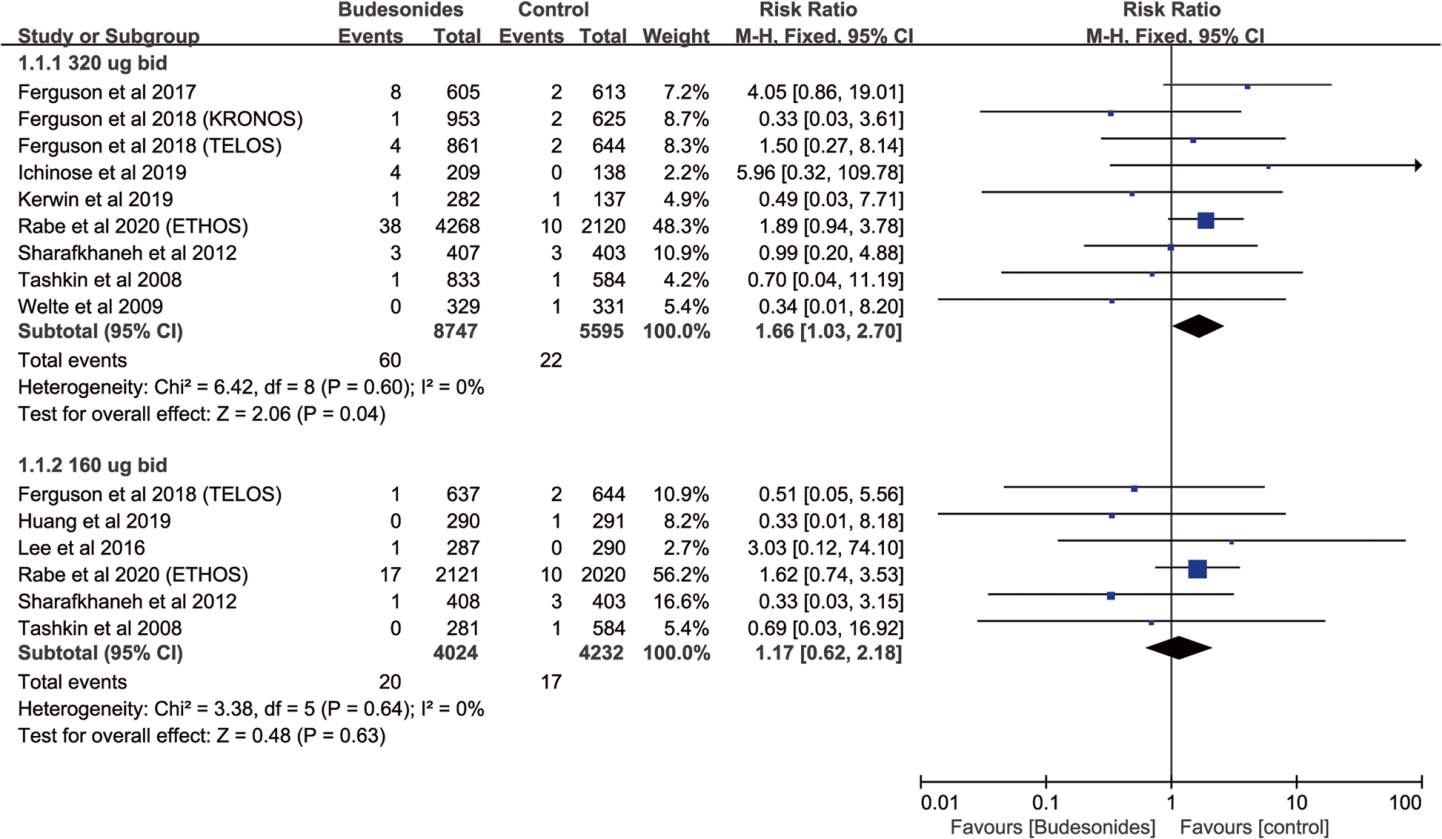
Risk of fractures with budesonides therapy vs. Inhaled therapy without ICSs according to different doses

**Fig. 5:**
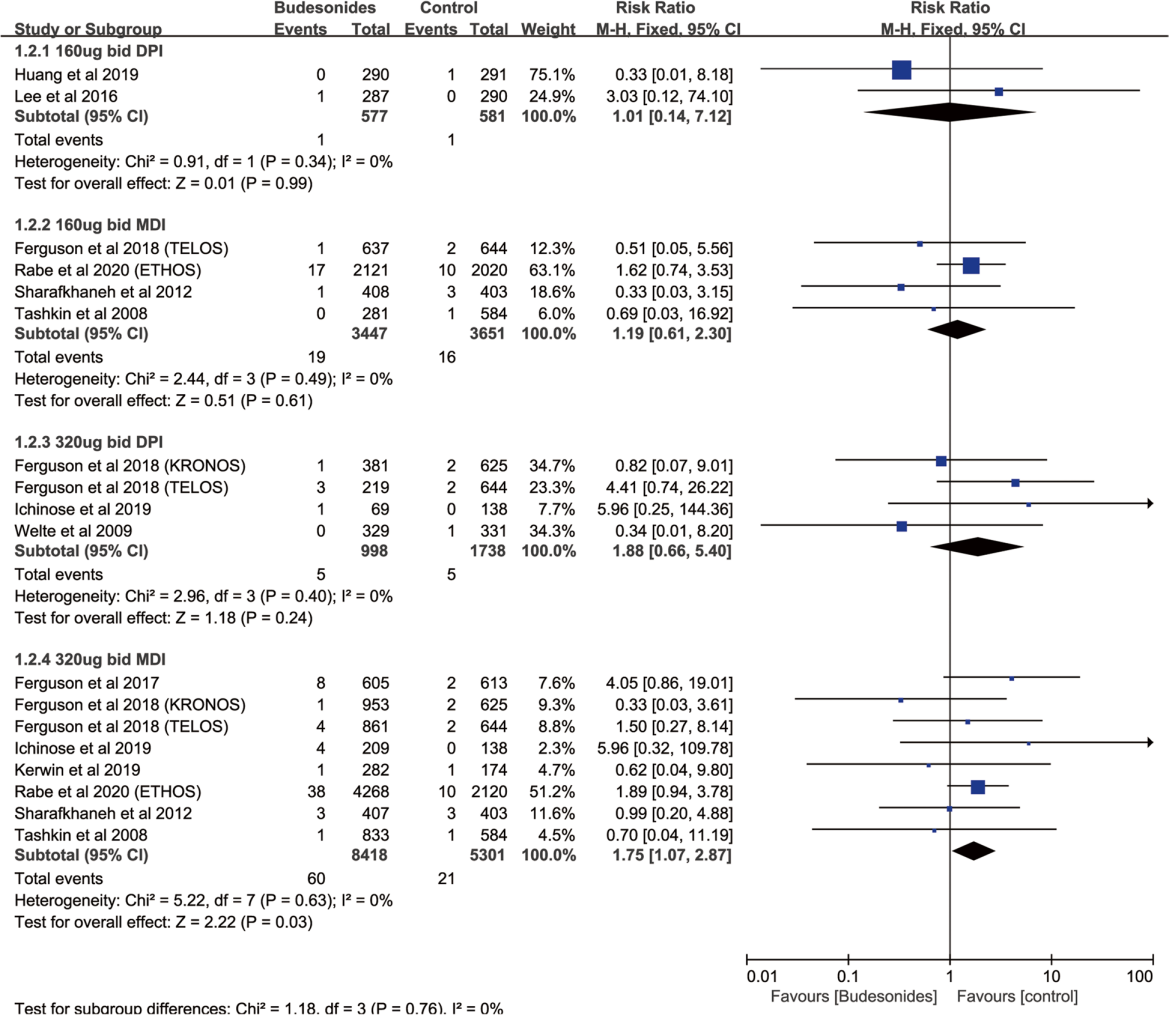
Risk of fractures with budesonides therapy vs. Inhaled therapy without ICSs according to different inhalation devices

Compared with control, subgroup analysis based on different types revealed that budesonide therapy (12 RCTs; RR, 1.64; 95%CI., 1.07–2.51; *P* = 0.02; heterogeneity: *I* ^2^ = 0) and fluticasone furoate therapy (13 RCTs; RR, 1.37; 95%CI, 1.05–1.78; *P* = 0.02; heterogeneity: *I* ^2^ = 0) was associated with a significantly increased the risk of fractures, but did not significantly increase the risk of fractures in patients who were on treatment with triamcinolone, mometasone furoate, fluticasone propionate or beclometasone dipropionate (Fig. 3). And budesonide 320 ug bid (9 RCTs; RR, 1.66; 95%CI., 1.03–2.70; *P* = 0.04; heterogeneity: *I* ^2^ = 0) was associated with a significantly increased the risk of fractures (Fig. 4). Fluticasone furoate 100 ug qd (11 RCTs; RR, 1.37; 95%CI, 1.04–1.80; *P* = 0.02; heterogeneity: *I* ^2^ = 0) was associated with a significantly increased the risk of fractures (Figure S9b). Grouping based on different inhalation devices, 320 ug bid budesonide via metered-dose inhaler (MDI) (8 RCTs; RR, 1.75; 95%CI, 1.07–2.87; *P* = 0.03; heterogeneity: *I* ^2^ = 0) was associated with a significantly increased risk of fractures while 320 ug bid budesonide via dry powder inhaler (DPI) (4 RCTs; RR, 1.88; 95%CI, 0.66–5.40; *P* = 0.24; heterogeneity: *I* ^2^ = 0) had no relationship with the increase of fracture risk (Fig. 5). Moreover, there was no difference in fracture risk between patients with different inhalation devices of fluticasone propionate and the control group (Figure S10). Because all the patients who were treated in fluticasone furoate were only absorbed by DPI inhalers, we didn't group them by inhalers.

**Fig. 6:**
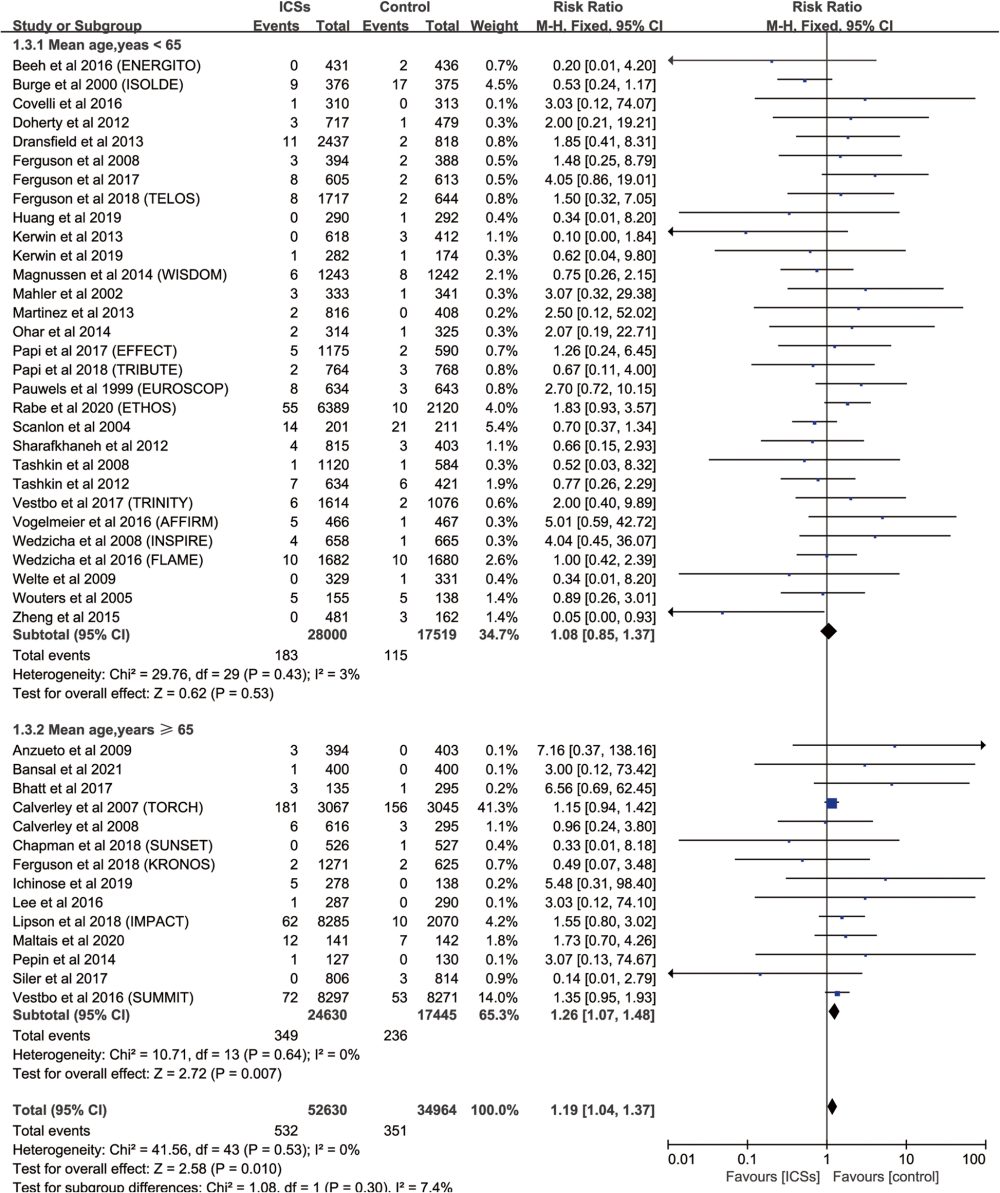
Risk of fractures with ICSs therapy vs. Inhaled therapy without ICSs in patients with different mean ages

Subgroup analysis based on mean age of patients revealed that mean age ≥ 65 (6 RCTs; RR, 1.27; 95%CI, 1.01–1.61; *P* = 0.04; heterogeneity: *I* ^2^ = 0) was associated with a significantly increased the risk of fractures compared with LABA in patients (Fig. 6).

**Fig. 7:**
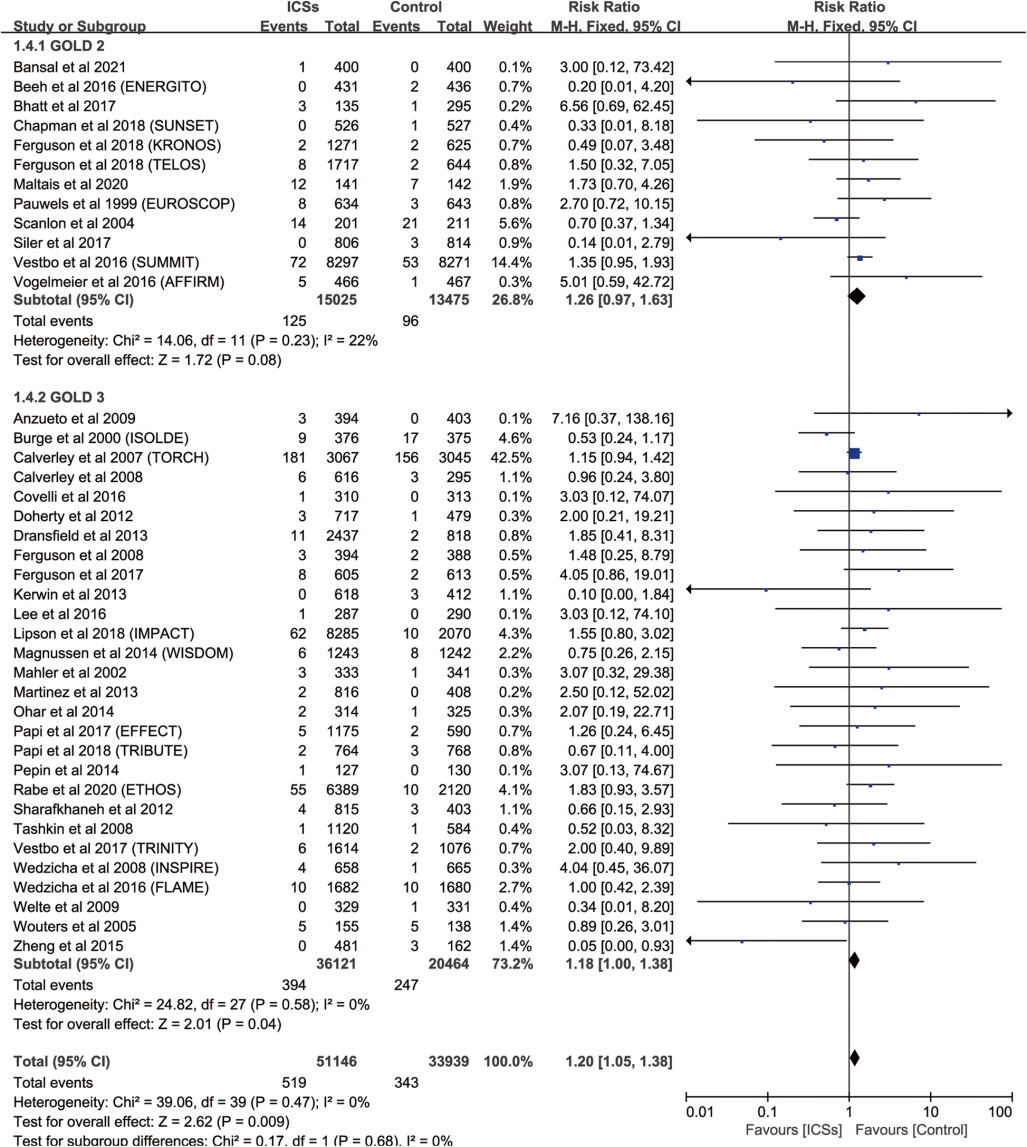
Risk of fractures with ICSs therapy vs. Inhaled therapy without ICSs in patients with different severities

Subgroup analysis based on GOLD grade revealed that GOLD 3 (28 RCTs; RR, 1.18; 95%CI, 1.00–1.38; *P* = 0.04; heterogeneity: *I* ^2^ = 0) was associated with a significantly increased the risk of fractures compared with control (Fig. 7).

### Risk of fractures with different ICSs therapy vs. Controls

**Fig. 8:**
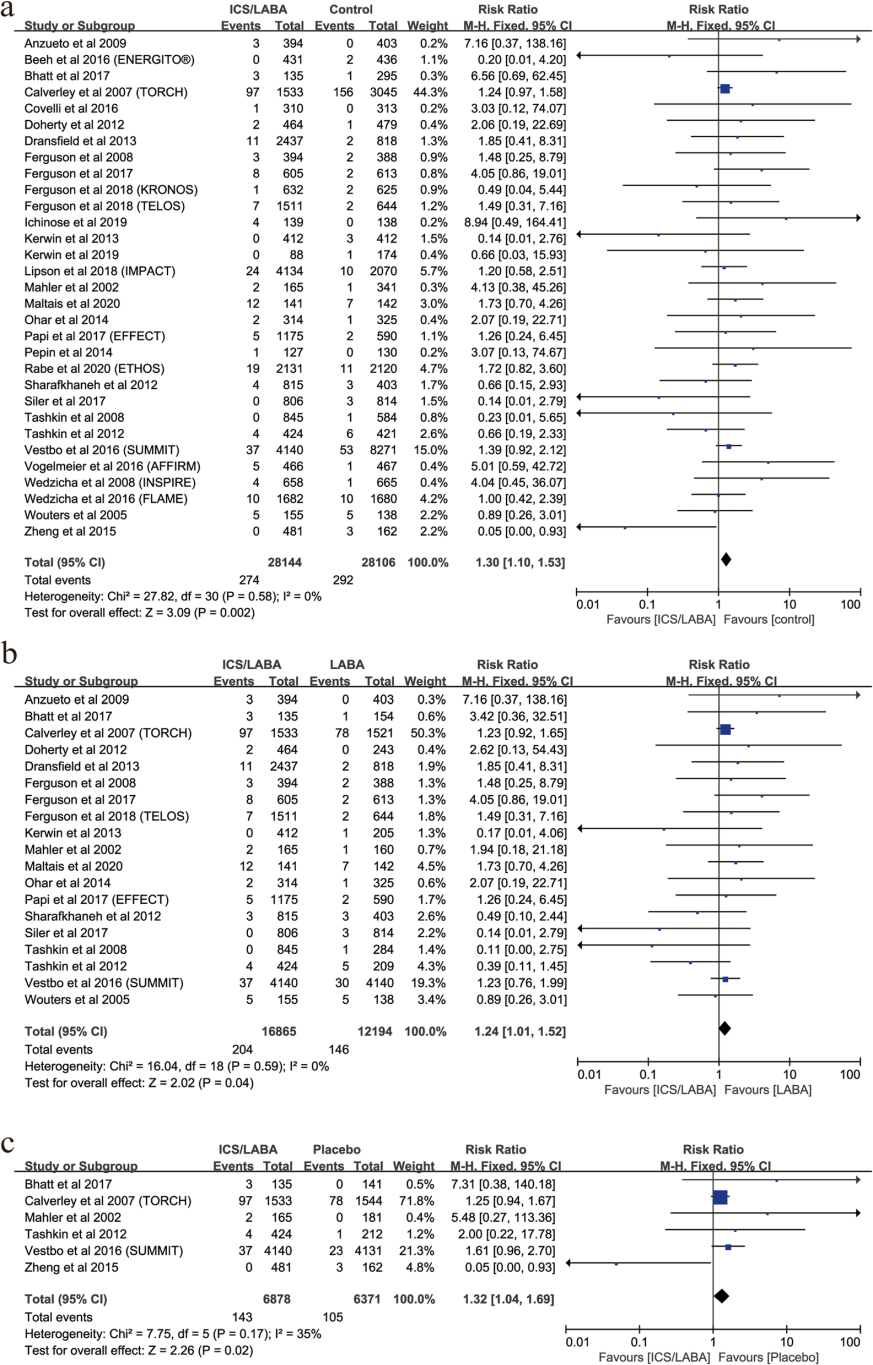
Risk of fractures with ICS/LABA therapy vs. Controls. (**a**: Risk of fractures with ICS/LABA therapy vs. Control; **b**: Risk of fractures with ICS/LABA therapy vs. LABA; **c**: Risk of fractures with ICS/LABA therapy vs. Placebo)

**Fig. 9:**
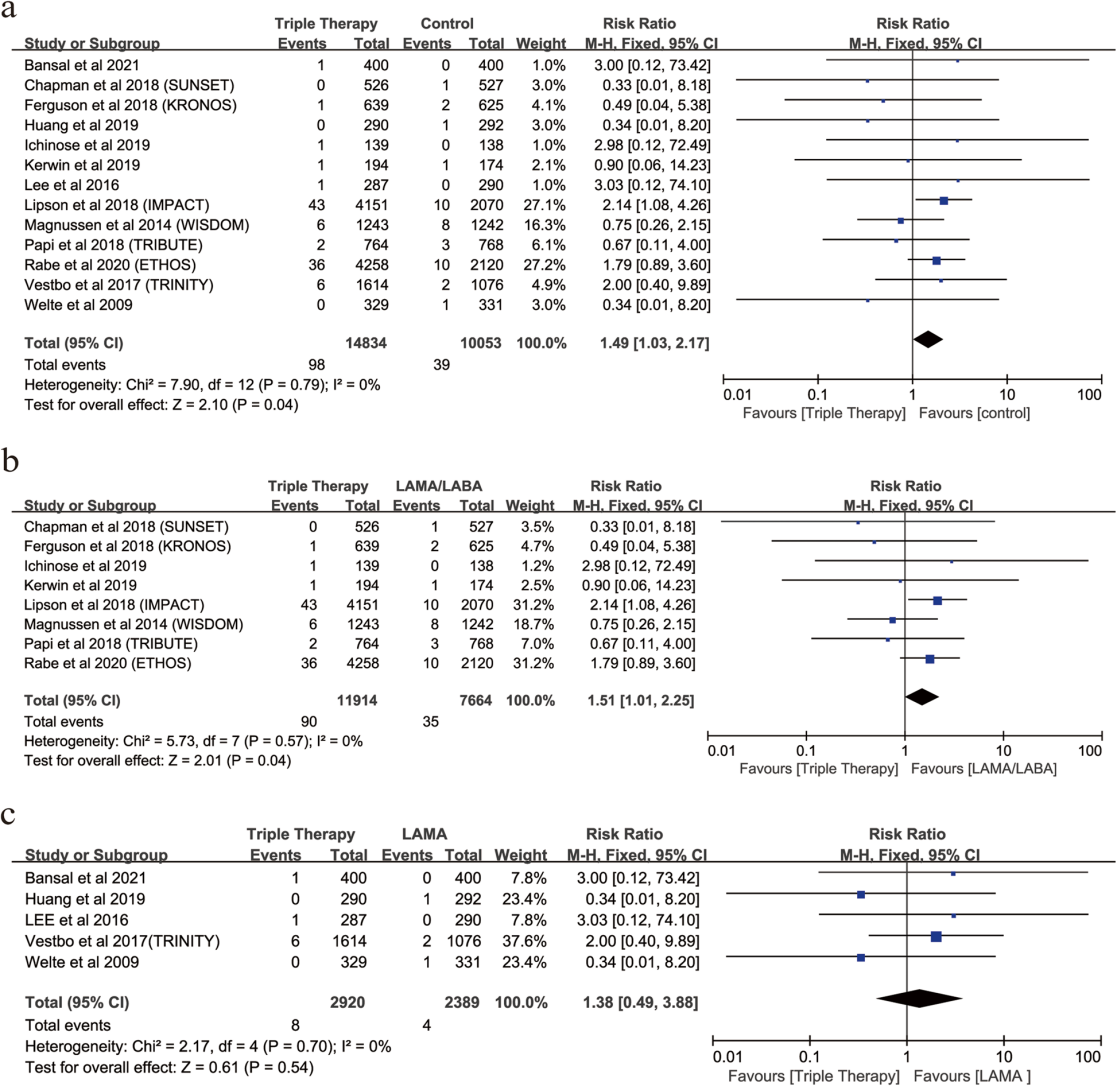
Risk of fractures with triple therapy vs. Controls. (**a**:Risk of fractures with triple therapy vs. Control; **b**: Risk of fractures with triple therapy vs. LAMA/LABA; **c**: Risk of fractures with triple therapy vs. LAMA)

Of the included RCTs, 31 RCTs (56,250 patients), 13 RCTs (24,887 patients), and 12 RCTs (17,557 patients) involved ICS/LABA, triple therapy, and mono-ICS therapy. ICS/LABA (RR, 1.30; 95%CI, 1.10–1.53; *P* = 0.002; heterogeneity: *I* ^2^ = 0) or triple therapy (RR, 1.49; 95%CI, 1.03–2.17; *P* = 0.04; heterogeneity: *I* ^2^ = 0), rather than mono-ICS therapy (RR, 1.07; 95%CI, 0.86–1.33; *P* = 0.52*;* heterogeneity: *I* ^2^ = 4), was associated with a significantly increased the risk of fractures in patients compared with controls (Figs. 8a and 9a, Figure S4).

### Risk of fractures with ICS/LABA vs. different controls

Of 31 RCTs for ICS/LABA therapy compared with controls, ICS/LABA compared with LABA (19 RCTs; RR, 1.24; 95%CI, 1.01–1.44; *P* = 0.04; heterogeneity: *I* ^2^ = 0) and ICS/LABA compared with placebo (6 RCTs; RR, 1.32; 95%CI, 1.04–1.69; *P* = 0.02; heterogeneity: *I* ^2^ = 35), rather than ICS/LABA compared with LAMA/LABA (8 RCTs; RR, 1.28; 95%CI, 0.94–2.10; *P* = 0.10; heterogeneity: *I* ^2^ = 0) or ICS/LABA compared with LAMA (3 RCTs: RR, 3.55; 95%CI, 0.74–17.03; *P* = 0.11; heterogeneity: *I* ^2^ = 0) were associated with a significantly increased the risk of fractures in patients compared with controls (Fig. 8b, c, Figure S5a, b).

### Subgroup analysis for risk of fractures with ICS/LABA vs. LABA

Subgroup analysis based on mean age of patients revealed that mean age ≥ 65 (6 RCTs; RR, 1.27; 95%CI, 1.01–1.61; *P* = 0.04; heterogeneity: *I* ^2^ = 0) was associated with a significantly increased the risk of fractures compared with LABA in patients (Figure S6b).

### Risk of fractures with triple therapy vs. different controls

Of 13 RCTs for triple therapy compared with controls, triple therapy compared with LAMA/LABA (8 RCTs; RR, 1.51; 95%CI, 1.01–2.25; *P* = 0.04; heterogeneity: *I* ^2^ = 0) rather than triple therapy compared with LAMA (5 RCTs; RR, 1.38; 95%CI, 0.49–3.88; *P* = 0.54; heterogeneity: *I* ^2^ = 0) was associated with a significantly increased the risk of fractures in patients compared with controls (Fig. 9b, c).

### Subgroup analysis for risk of fractures with triple therapy vs. LAMA/LABA

**Fig. 10:**
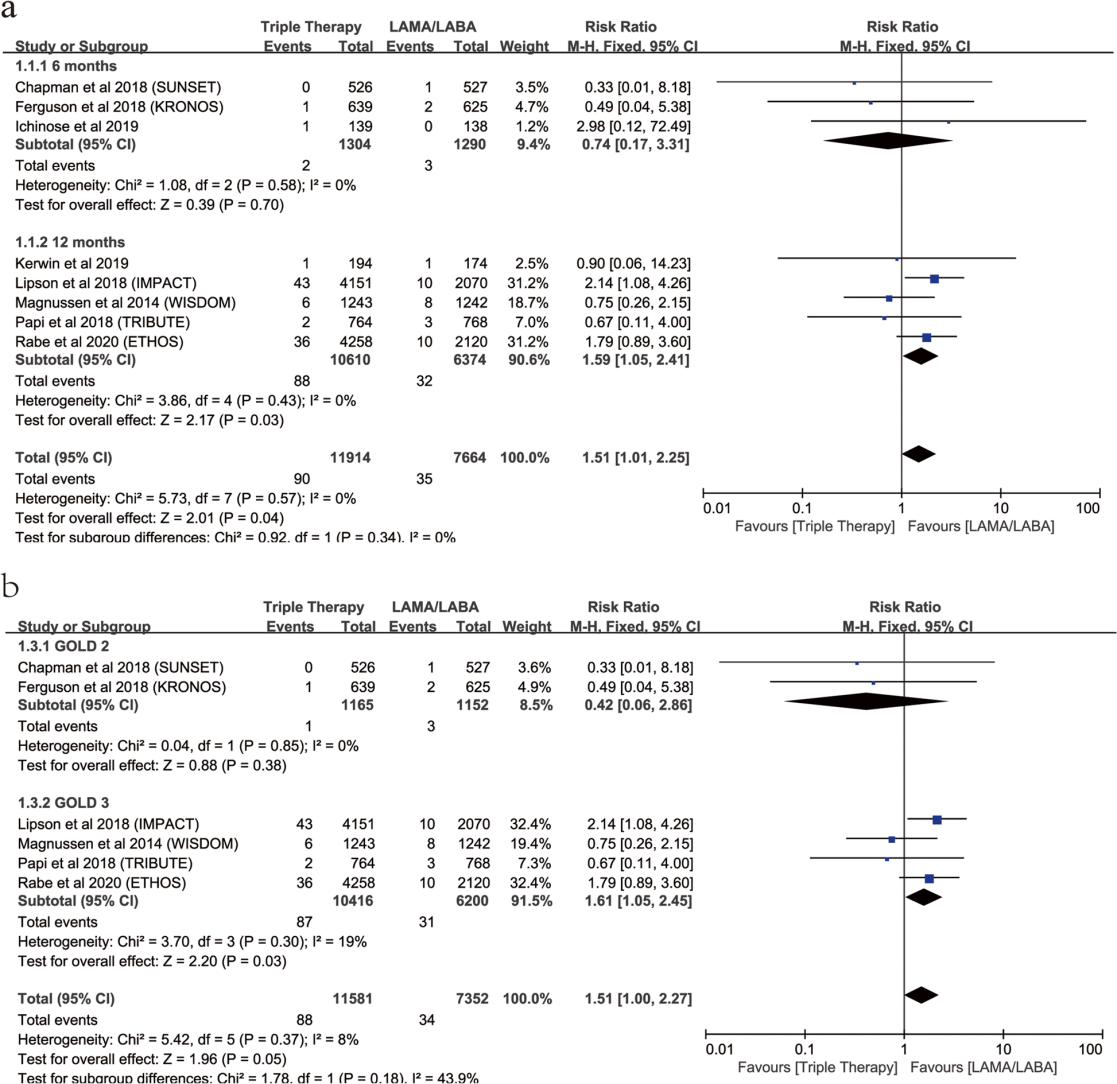
Subgroup analysis of risk of fractures with triple therapy vs. LAMA/LABA (**a**: Risk of fractures with triple therapy vs. LAMA/LABA based on duration; **b**: Risk of fractures with triple therapy vs. LAMA/LABA based on GOLD grade)

Subgroup analysis based on duration of follow-up revealed that triple therapy (5 RCTs; RR, 1.59; 95%CI, 1.05–2.41; *P* = 0.03; heterogeneity: *I* ^2^ = 0) was associated with a significantly increased the risk of fractures compared with LAMA/LABA in patients who continue the treatment for at least 12 months (Fig. 10a).

Subgroup analysis based on GOLD grade revealed that GOLD 3 (4 RCTs; RR, 1.61; 95%CI, 1.05–2.45; *P* = 0.03; heterogeneity: *I* ^2^ = 19%) was associated with a significantly increased the risk of fractures compared with LAMA/LABA (Fig. 10b).

### Sensitivity analyses

When using the Mantel–Haenszel method to calculate risk ratios with the fixed-effect model, the results of the sensitivity analysis showed that four large RCTs (the TORCH trail, the SUMMIT trail, the IMPACT trail, and the ETHOS trail) accounted for a large proportion of effect on the overall effect, and two RCTs (the ISOLDE trail and the study of Scanlon et al. [44]) that reported too many fracture events in the control group compared with ICSs group also had an effect on the pooled results (Figure S7). However, excluding any one result of 44 RCTs did not significantly alter the pooled results or any heterogeneity.

## Discussion

In this systematic review and meta-analysis based on 44 randomized controlled trials (87,594 patients), we found that compared with that without ICSs, inhalation therapy with ICSs was associated with increased risks of fracture. Considering that due to the inclusion of different types and doses of ICS, the above pooled results may not avoid heterogeneity, and then we conduct subgroup analyses. Subgroup analysis showed that the predictors of this association were treatment duration of ≥ 12 months, budesonide therapy, or fluticasone furoate therapy. According to the dosage and inhalation device, we found that budesonide of 320ug bid and MDI inhalation device was related to the increased risk of fracture. But fluticasone furoate and fluticasone propionate in different inhalation devices have no relationship with the increase in fracture risk. ICS/LABA combined therapy and triple therapy were significantly related to the fracture risk of COPD patients compared with no ICS therapy, while ICS alone has no significant relationship with the increase of fracture risk compared with the placebo group. Compared with LABA alone, further subgroup analysis of ICS/LABA group showed that the subgroup with the average age ≥ 65 had a significant correlation with the increased risk of fracture. Compared with LAMA/LABA combined therapy, the further subgroup analysis of triple therapy group showed that the subgroup with GOLD grade of GOLD 3 was significantly related to the increased fracture risk of patients after treatment for more than 12 months.

The exact mechanisms by which ICSs increase the risk of fracture in COPD patients are unclear. However, due to malnutrition, inflammatory response, and previous exposure to corticosteroids, COPD patients are at risk of fracture porosity and fracture [58]. Long-term and intensive ICS therapy may lead to a small part being absorbed and have systemic effects [59], resulting in increased bone absorption and decreased bone formation. Moreover, osteoporosis is an important complication of COPD. With the growth of age, the loss of bone density will become more and more serious [60]. However, most COPD patients are elderly, and age is also an independent risk factor for COPD [61]. Taken together, these factors seem to amplify the influence of ICS on the fracture risk of the COPD population.

Previous systematic reviews have shown that ICSs are not associated with fracture risk in patients with COPD [62–65]. However, these results appear to be controversial because of the earlier and fewer articles included. Our results are consistent with those of another systematic review, where ICSs treatment duration of ≥ 12 months, budesonide therapy, or fluticasone furoate therapy increases the risk of fracture in patients with COPD [9]. Compared with this previous systematic review and meta-analyses [9], we conducted a more comprehensive search, including more randomized controlled trials and a larger sample size. The ARCTIC study, a large-scale cohort study in Sweden based on ICSs and the risk of osteoporosis and fracture, shows that the risk of fracture of ICSs is dose-dependent, and the risk of fracture is associated with the risk of osteoporosis [10]. This is consistent with our results, which showed a significant association between higher doses of budesonide (≥ 320 ug bid) and an increased risk of fracture. In addition, some studies have shown that different inhalation devices are ultimately related to different lung deposition and absorption [66]. Our results showed that, compared with control groups, 320 ug bid budesonide via MDI was significantly associated with an increased risk of fracture while 320 ug bid budesonide via DPI was not associated with an increased risk of fracture. It seems that different inhalation devices have different effects on the fracture risk of ICSs treatment. Several researchers found that fluticasone furoate had a greater potency than other ICSs [67, 68]. Our results showed that fluticasone furoate was significantly associated with an increased risk of fracture. Due to fewer studies and samples included in the 200 ug bid fluticasone group, it did not show a dose-dependent relationship. In a post-hoc analysis based on the TORCH study, there were no significant differences in BMD between the ICS/LABA (SAL/FP) and LABA (SAL) alone [69]. However, our results show that ICS/LABA is significantly associated with an increased risk of fracture in patients with COPD compared with LABA. The ETHOS study results showed no differences in fracture risk between triple therapy and LAMA/LABA [54]. However, our combined results from eight randomized controlled trials showed that triple therapy significantly increased the risk of fracture in patients with COPD. In addition, subgroup analyses based on the baseline characteristics of patients showed that patients with COPD with a mean age greater than 65 years and GOLD 3 were significantly associated with an increased risk of fracture. Older people were usually associated with increased risks of osteoporosis and fractures [11]. Similarly, GOLD 3 OPD subjects were usually older and sedentary. Both age and disease severity contributed to the increased risks of osteoporosis and fracture.

There was no increase in fracture risk with ICS alone compared to the placebo control group. This result should be interpreted cautiously. Some studies that included ICS and placebo did not report fracture events. Moreover, in the ISOLDE trial [48] in 2000 and the study by Scanlon et al [44] in 2004, too many fracture events were reported in the placebo therapy group compared with the ICS therapy group. In addition, inhaled bronchodilators may have a synergistic effect on inhaled glucocorticoids, and inhaled bronchodilators amplify the effect of inhaled glucocorticoids [70]. A previous clinical study found that inhaled long-acting β_2_-agonists enhanced glucocorticoid receptor nuclear translocation in patients with COPD [71]. These may be reasons why the risk of fractures was significantly associated with the ICS combination therapy compared with the non-ICS inhalation therapy.

## Limitations and Strengths

There are some limitations in our thesis. First, RCTs with different complications might have different effects on fracture risk, and our study did not consider the baseline complications of RCTs. Second, RCTs with different medical histories might also lead to different fracture risks. Third, We did not classify different fractures. Perhaps specific ICSs treatment is associated with an increased risk of specific fracture types. Finally, manual retrieval inevitably produced publication bias, although the Egger test and Begg test did not show publication bias.

Despite these limitations, our study is of great clinical significance to the current work. First, as far as we know, this paper is the largest meta-analysis of randomized controlled trials so far, which comprehensively evaluated the fracture risks related to ICSs treatment. Second, fracture and osteoporosis are common complications of COPD, and ICS inhalation therapy is a commonly used drug to prevent and alleviate the acute attack of COPD patients. At present, the two require higher evidence-based medical shreds of evidence to establish the connection. However, several large-scale randomized controlled trials have failed to solve the problem directly. Against this background, our results demonstrate that ICS inhalation therapy, especially ICS/LABA and triple therapy, significantly increased the risk of fracture in COPD patients. Third, most RCTs exclude patients with severe fracture porosity and fractures, and some RCTs do not report fracture events. Therefore, the impact of ICSs on fracture risk in patients with COPD may be significantly greater in the real-world environment than in RCTs.

## Conclusions

Inhalation therapy containing ICS, especially ICS/LABA and triple therapy, significantly increases the fracture risk of patients with chronic obstructive pulmonary disease compared with non-ICS inhalation therapy. Treatment duration ≥ 12 months, mean age of study participants ≥ 65 months, and GOLD stage III were significantly associated with an increased risk of fracture. In addition, budesonide and fluticasone furoate were associated with this risk. Budesonide in high doses and via MDI was significantly associated with an increased risk of fracture. However, the excess risk of fracture should be balanced against their benefits. ICS/LABA or triple therapy can improve the patient's condition, reduce the frequency of aggravated hospitalization, and improve the quality of life of patients. Therefore, for elderly patients with severe COPD requiring long-term ICSs therapy the application of ICSs in the treatment requires clinicians to weigh the advantages and disadvantages to prevent excessive use of ICSs.

### Supplementary Information

**Additional file 1: Table S1. **Search strategy.

**Additional file 2: Table S2.** Baseline Characteristics.

**Additional file 3: Table S3.** GRADE summary of findings.

**Additional file 4: Figure S1.** The result of the bias assessment: a: Risk of bias summary; b: Risk of bias graph.

**Additional file 5: Figure S2.** The approximate symmetry in the funnel plot of publication bias.

**Additional file 6**:** Figure S3.** The results from the Egger test and Begg test: a) Begg’ s test publication plot; b)Egger’s test publication plot; c) Test of publication bias of Begg’ s test and Egger’ s test).

**Additional file 7: Figure S4.** Riskof fractures with mono-ICS therapy vs. Placebo.

**Additional file 8: Figure S5.** Risk of fractures with ICS/LABA therapy vs. Controls: a) Risk of fractures with ICS/LABA therapy vs. LAMA/LABA; b) Risk of fractures with ICS/LABA therapy vs. LAMA.

**Additional file 9: Figure S6.** Subgroup analysis of risk of fractures with ICS/LABA vs. LABA: a) Risk of fractures with ICS/LABA therapy vs. LABA based on duration; b) Risk of fractures with ICS/LABA therapy vs. LABA based on mean age; c) Risk of fractures with ICS/LABA therapy vs. LABA based on GOLD grade.

**Additional file 10: Figure S7.** The result of the sensitivity analyses.

**Additional file 11: Figure S8.** Risk of fractures with ICSs therapy vs. Inhaled therapy without ICSs according to different treatment duration.

**Additional file 12: Figure S9.** a. Risk of fractures with fluticasone propionate vs. control according to different doses; b. Risk of fractures with fluticasone propionate vs. control according to different doses.

**Additional file 13:** **Figure S10.** Risk of fractures with fluticasone propionate vs. control according to different inhalation device.

## Data Availability

All datasets generated and analysed during this study are included in this published article and its supplementary information files.

## Abbreviations

COPD: Chronic obstructive pulmonary disease
GOLD: Global Initiative for Chronic Obstructive Pulmonary Disease
ICS: Inhaled corticosteroid
LAMA: Long-acting muscarinic antagonists
LABA: Long-acting β_2_-agonists
PRISMA: Preferred Reporting Items for System Review and Meta-Analysis
RCT: Randomized controlled study
RR: Risk ratio
CI: Confidence interval
MDI: Metered-dose inhaler
DPI: Dry powder inhaler
