# Supplementary material for: Effect of fracture risk in inhaled corticosteroids in patients with chronic obstructive pulmonary disease: a systematic review and meta-analysis
Source: BMC Pulm Med. 2023 Aug 17;23:304. doi: 10.1186/s12890-023-02602-5 (PMC10436625; doi:10.1186/s12890-023-02602-5)
Supplement: Supplementary file 1 — Additional file 1: Table S1. Search strategy. [file 12890_2023_2602_MOESM1_ESM.docx]

**Table S1:** Search strategy

| Databases | Terms Searched |
| --- | --- |
| General search strategy | ((Pulmonary Disease, Chronic Obstructive[MeSH Terms]) OR (Chronic Obstructive Lung Disease) OR (Chronic Obstructive Pulmonary Diseases) OR (COPD) OR (Chronic Obstructive Airway Disease) OR (Chronic Obstructive Pulmonary Disease) OR (Airflow Obstruction, Chronic) OR (Airflow Obstructions, Chronic) OR (Chronic Airflow Obstructions) OR (Chronic Airflow Obstruction) and ((ICS) OR (inhaled corticosteroids) OR (Inhaled glucocorticoids) OR (fluticasone) OR (flunisolide) OR (budesonide) OR (beclomethasone) OR (triamcinolone) OR (mometasone) OR (ciclesonide)) with the clinical trial filters (Clinical Trial, Humans, English) |
| PubMed | #1 (Pulmonary Disease, Chronic Obstructive[MeSH Terms]) OR (Chronic Obstructive Lung Disease) OR (Chronic Obstructive Pulmonary Diseases) OR (COPD) OR (Chronic Obstructive Airway Disease) OR (Chronic Obstructive Pulmonary Disease) OR (Airflow Obstruction, Chronic) OR (Airflow Obstructions, Chronic) OR (Chronic Airflow Obstructions) OR (Chronic Airflow Obstruction) |
|  | #2 (ICS) OR (inhaled corticosteroids) OR (Inhaled glucocorticoids) OR (fluticasone) OR (flunisolide) OR (budesonide) OR (beclomethasone) OR (triamcinolone) OR (mometasone) OR (ciclesonide) |
|  | #3 (Randomized Controlled Trial) OR (RCT) |
|  | #1 and #2 and #3 |
| ENDBASE | #1 ('chronic obstructive lung disease'/exp OR 'chronic obstructive lung disease' OR (chronic AND obstructive AND ('lung'/exp OR lung) AND ('disease'/exp OR disease)) OR 'chronic airflow obstruction'/exp OR 'chronic airflow obstruction' OR (chronic AND ('airflow'/exp OR airflow) AND ('obstruction'/exp OR obstruction)) OR 'chronic airway obstruction'/exp OR 'chronic airway obstruction' OR (chronic AND ('airway'/exp OR airway) AND ('obstruction'/exp OR obstruction)) OR 'chronic obstructive bronchopulmonary disease'/exp OR 'chronic obstructive bronchopulmonary disease' OR (chronic AND obstructive AND bronchopulmonary AND ('disease'/exp OR disease)) OR 'chronic obstructive lung disorder'/exp OR 'chronic obstructive lung disorder' OR (chronic AND obstructive AND ('lung'/exp OR lung) AND ('disorder'/exp OR disorder)) OR 'chronic obstructive pulmonary disease'/exp OR 'chronic obstructive pulmonary disease' OR (chronic AND obstructive AND pulmonary AND ('disease'/exp OR disease)) OR 'chronic obstructive pulmonary disorder'/exp OR 'chronic obstructive pulmonary disorder' OR (chronic AND obstructive AND pulmonary AND ('disorder'/exp OR disorder)) OR 'chronic obstructive respiratory disease'/exp OR 'chronic obstructive respiratory disease' OR (chronic AND obstructive AND ('respiratory'/exp OR respiratory) AND ('disease'/exp OR disease)) OR 'chronic pulmonary obstructive disease'/exp OR 'chronic pulmonary obstructive disease' OR (chronic AND pulmonary AND obstructive AND ('disease'/exp OR disease)) OR 'chronic pulmonary obstructive disorder'/exp OR 'chronic pulmonary obstructive disorder' OR (chronic AND pulmonary AND obstructive AND ('disorder'/exp OR disorder)) OR 'copd'/exp OR copd OR 'lung chronic obstructive disease'/exp OR 'lung chronic obstructive disease' OR (('lung'/exp OR lung) AND chronic AND obstructive AND ('disease'/exp OR disease)) OR 'lung disease, chronic obstructive'/exp OR 'lung disease, chronic obstructive' OR (('lung'/exp OR lung) AND ('disease,'/exp OR disease,) AND chronic AND obstructive) OR 'obstructive chronic lung disease'/exp OR 'obstructive chronic lung disease' OR (obstructive AND chronic AND ('lung'/exp OR lung) AND ('disease'/exp OR disease)) OR 'obstructive chronic pulmonary disease'/exp OR 'obstructive chronic pulmonary disease' OR (obstructive AND chronic AND pulmonary AND ('disease'/exp OR disease)) OR 'obstructive lung disease, chronic'/exp OR 'obstructive lung disease, chronic' OR (obstructive AND ('lung'/exp OR lung) AND ('disease,'/exp OR disease,) AND chronic) OR 'pulmonary disease, chronic obstructive'/exp OR 'pulmonary disease, chronic obstructive' OR (pulmonary AND ('disease,'/exp OR disease,) AND chronic AND obstructive) OR 'pulmonary disorder, chronic obstructive'/exp OR 'pulmonary disorder, chronic obstructive' OR (pulmonary AND ('disorder,'/exp OR disorder,) AND chronic AND obstructive)) |
|  | #2 ('ICS' OR 'inhaled corticosteroids' OR 'fluticasone'/exp OR 'fluticasone' OR 'flunisolide'/exp OR 'flunisolide' OR 'budesonide'/exp OR 'budesonide' OR 'beclomethasone'/exp OR 'beclomethasone' OR 'triamcinolone'/exp OR 'triamcinolone' OR 'mometasone'/exp OR 'mometasone' OR 'ciclesonide'/exp OR 'ciclesonide') |
|  | #3 ('randomized controlled trial'/exp OR 'randomized controlled trial' OR (randomized AND controlled AND ('trial'/exp OR trial) |
|  | #1 and #2 and #3 |
| Cochrane Library | #1 (Pulmonary Disease, Chronic Obstructive) OR (Chronic Obstructive Airway Disease) OR (Chronic Obstructive Lung Disease) OR (COPD) OR (Chronic Obstructive Pulmonary Diseases) OR (Chronic Obstructive Pulmonary Disease) OR (Airflow Obstruction, Chronic) OR (Chronic Airflow Obstructions) OR (Chronic Airflow Obstruction) OR (Airflow Obstructions, Chronic) |
|  | #2 (ICS) OR (inhaled corticosteroids) OR (Inhaled glucocorticoids) OR (fluticasone) OR (flunisolide) OR (budesonide) OR (beclomethasone) OR (triamcinolone) OR (mometasone) OR (ciclesonide) |
|  | #3 “tria” |
|  | #1 and #2 and #3 |
| Web of Science | #1 (TS=(Pulmonary Disease, Chronic Obstructive OR Chronic Obstructive Lung Disease OR Chronic Obstructive Pulmonary Diseases OR COPD OR Chronic Obstructive Airway Disease OR Chronic Obstructive Pulmonary Disease OR Airflow Obstruction, Chronic OR Airflow Obstructions, Chronic OR Chronic Airflow Obstructions OR Chronic Airflow Obstruction ) |
|  | #2 TS=(ICS OR inhaled corticosteroids OR Inhaled glucocorticoids OR fluticasone OR flunisolide OR budesonide OR beclomethasone OR triamcinolone OR mometasone OR ciclesonide) |
|  | #3 TS=(Randomized Controlled Trial OR RCT) |
|  | #1 and #2 and #3 |
| MEDLINE | ( ((Pulmonary Disease, Chronic Obstructive) OR (Chronic Obstructive Airway Disease) OR (Chronic Obstructive Lung Disease) OR (COPD) OR (Chronic Obstructive Pulmonary Diseases) OR (Chronic Obstructive Pulmonary Disease) OR (Airflow Obstruction, Chronic) OR (Chronic Airflow Obstructions) OR (Chronic Airflow Obstruction) OR (Airflow Obstructions, Chronic)) ) AND ( ((ICS) OR (inhaled corticosteroids) OR (Inhaled glucocorticoids) OR (fluticasone) OR (flunisolide) OR (budesonide) OR (beclomethasone) OR (triamcinolone) OR (mometasone) OR (ciclesonide)) ) AND ( (Randomized Controlled Trial OR RCT) ) |
