# Supplementary material for: Effect of fracture risk in inhaled corticosteroids in patients with chronic obstructive pulmonary disease: a systematic review and meta-analysis
Source: BMC Pulm Med. 2023 Aug 17;23:304. doi: 10.1186/s12890-023-02602-5 (PMC10436625; doi:10.1186/s12890-023-02602-5)
Supplement: Supplementary file 2 — Additional file 2: Table S2. Baseline Characteristics. [file 12890_2023_2602_MOESM2_ESM.docx]

**TABLE S2: Baseline Characteristics**

| **Interventions/Control** | **Fracture events, N** | **No. Of Participants (%Male)** | **Age, Mean (SD), y** | **% Predicted FEV1, Mean (SD)** | **Current**  **Smokers,**  **(N%)** | Duration, months |
| --- | --- | --- | --- | --- | --- | --- |
| **Papi et al 2018 (TRIBUTE)**  **(NCT02579850)** |  |  |  |  |  |  |
| BDP/FF/G(87 ug/5 ug/9 ug) | 2 | 764（72%） | 64·4 (7·7) | 36.4 (8.0) | 351 (46%) | 12 |
| IND/GLY (85 ug/43 ug) | 3 | 768（72%） | 64·5 (7·7) | 36·4 (8·1) | 332 (43%) |  |
| **Sharafkhaneh et al 2012**  **(NCT00419744)** |  |  |  |  |  |  |
| BUD/FM pMDI 320/9 ug bid | 3 | 407(64.4) | 63.8 (9.4) | 37.9 (11.8) | 138(33.9) | 12 |
| BUD/FM pMDI 160/9 ug bid | 1 | 408(64.7) | 62.8 (9.2) | 37.6 (11.6) | 143 (35.1) |  |
| FM DPI 9 ug bid | 3 | 403(56.8) | 62.5 (9.4) | 37.5 (12.4) | 154 (38.2) |  |
| **Anzueto et al 2009**  **(NCT00115492)** |  |  |  |  |  |  |
| FSC 250ug/50ug | 3 | 394(51) | 65.4 ± 9.1 | 41.2± 14.3 | 42 | 12 |
| SAL 50ug | 0 | 403(57) | 65.3 ± 8.8 | 40.0 ± 12.6 | 43 |  |
| **Papi et al 2017 (EFFECT)**  **(EudraCT 2012–004162–17)** |  |  |  |  |  |  |
| FP/FORM 500/20ug bid | 2 | 587(75.5) | 63.8 (7.92) | 37.8 (7.88) | 45.7 | 12 |
| FP/FORM 250/10 ug bid | 3 | 588(72.6) | 63.0 (7.81) | 38.0 (7.74) | 49.1 |  |
| FORM 12 ug bid | 2 | 590(75.9) | 64.0 (7.87) | 37.7 (7.95) | 50 |  |
| **Vogelmeier et al 2016 (AFFIRM)**  **(NCT01908140)** |  |  |  |  |  |  |
| salmeterol/fluticasone 50/500 µg | 5 | 466(64.4%) | 63.3±7.5 | 53.2±14.8 | NA | 6 |
| aclidinium/formoterol 400/12 µg | 1 | 467(65.7%) | 63.5±8.1 | 53.3±14.4 | NA |  |
| **Lipson et al 2018 (IMPACT)**  **(NCT02164513)** |  |  |  |  |  |  |
| FF/UMEC/VI100 µcg/62.5µcg/25µcg qd DPI | 43 | 4151(67) | 65.3±8.2 | 45.7±15.0 | 35 | 12 |
| FF/VI 100ucg/25ucg QD via DPI | 24 | 4134(66) | 65.3±8.3 | 45.5±14.8 | 34 |  |
| UMEC/VI 62.5ucg/25ucg QD via DPI | 10 | 2070(66) | 65.2±8.3 | 45.4±14.7 | 35 |  |
| **Doherty et al 2012**  **(NCT00383721)** |  |  |  |  |  |  |
| MF/F 400/10 ug BID | 0 | 225(75) | 59.2 ± 9.1 | 38.1 ± 10.8 | 127 (56) | 12 |
| MF/F 200/10 ug BID | 2 | 239 (73) | 60.1 ± 9.0 | 38.7 ± 11.6 | 119 (50) |  |
| MF 400 ug BID | 1 | 253(78) | 60.5 ± 8.5 | 40.2 ± 11.7 | 134 (53) |  |
| F 10 ug BID | 0 | 243(75) | 59.7 ± 8.7 | 38.2 ± 12.3 | 123 (51) |  |
| Placebo | 1 | 236(75) | 58.8 ± 9.5 | 38.0 ± 11.5 | 120 (51) |  |
| **Mahler et al 2002**  **(SFCA3006)** |  |  |  |  |  |  |
| FSC | 2 | 165(62) | 61.9 | 41 | 76 (46) | 6 |
| F 500 ug | 1 | 168(61) | 64.4 | 41 | 77 (46) |  |
| S 50 ug | 1 | 160(64) | 63.5 | 40 | 74 (46) |  |
| Placebo | 0 | 181(75) | 64 | 41 | 97 (54) |  |
| **Tashkin et al 2008**  **(NCT00206154)** |  |  |  |  |  |  |
| BUD/FM pMDI 320/9 μg | 0 | 277(67.9) | 63.1 (9.0) | 39.05 (11.78) | 123 (44.4) | 6 |
| BUD/FM pMDI 160/9 μg | 0 | 281(64.4) | 63.6 (9.0) | 39.87 (11.23) | 126 (44.8) |  |
| BUD pMDI 320 ug FM DPI 9 μg | 0 | 287(74.2) | 63.7 (9.0) | 39.15 (11.41) | 119 (41.5) |  |
| BUD pMDI 320 μg | 1 | 275(67.6) | 63.4 (8.8) | 39.72 (12.01) | 118 (42.9) |  |
| FM DPI 9 μg | 1 | 284(65.5) | 63.5 (9.5) | 39.59 (12.76) | 119 (41.9) |  |
| PL | 0 | 300(69.0) | 63.2 (9.6) | 41.28 (12.14) | 119 (39.7) |  |
| **Tashkin et al 2012**  **(NCT00383435)** |  |  |  |  |  |  |
| MF/F 200/10 ug bid | 3 | 207(78) | 60.9 (8.1) | NA | 89 (43) | 12 |
| MF/F 400/10 ug bid | 1 | 217(79) | 59.7 (9.1) | NA | 99 (46) |  |
| MF 400 ug bid | 3 | 210(78) | 59.8 (8.9) | NA | 92 (44) |  |
| F 10 ug bid | 5 | 209(73) | 59.6 (8.5) | NA | 92 (44) |  |
| Placebo | 1 | 212(80) | 58.8 (8.7) | NA | 97 (46) |  |
| **Kerwin et al 2013**  **(NCT01053988)** |  |  |  |  |  |  |
| FF/VI 100/25 ug | 0 | 206(67) | 62.3 (8.49) | 47.8 (12.28) | 111 (54) | 6 |
| FF/VI 50/25 ug | 0 | 206(66) | 62.8 (9.13) | 48.4 (12.66) | 111 (54) |  |
| VI 25 ug | 1 | 205(68) | 63.4 (9.58) | 49.9 (12.05) | 111 (54) |  |
| FF 100 ug | 0 | 206(64) | 62.7 (9.47) | 46.9 (12.73) | 111 (54) |  |
| Placebo | 2 | 207(68) | 62.1 (8.80) | 48.5 (12.46) | 112 (54) |  |
| **Kerwin et al 2019**  **(NCT02536508)** |  |  |  |  |  |  |
| BGF MDI 320/18/9.6 μg | 1 | 194(52.6) | 62.6 (7.9) | NA | 101 (52.1) | 12 |
| BFF MDI 320/9.6 μg | 0 | 88 (60.2) | 64.0 (7.2) | NA | 42 (47.7) |  |
| GFF MDI 18/9.6 μg | 1 | 174 (50.0) | 62.4 (7.8) | NA | 95 (54.6) |  |
| **Wouters et al 2005 (COSMIC)** |  |  |  |  |  |  |
| Salmeterol + fluticasone 50ug/500ug BD | 5 | 189 (73) | 63.0 (7.9) | 47.4 (13.9) | 74 (39) | 12 |
| Salmetero 50ug BD | 5 | 184 (75) | 64.0 (7.7) | 48.2 (12.9) | 64 (35) |  |
| **Martinez et al 2013**  **(NCT01054885)** |  |  |  |  |  |  |
| FF/VI 200/25 ug DPI | 0 | 205(67) | 61.1 (8.58) | 47.1 (12.76) | 112 (55) | 6 |
| FF/VI 100/25 ug DPI | 0 | 204(71) | 61.9 (8.79) | 48.1 (12.85) | 109 (53) |  |
| VI 25 ug DPI | 0 | 203 (74) | 61.2 (8.62) | 48.5 (12.89) | 111 (55) |  |
| FF 200 ug DPI | 2 | 203(74) | 61.8 (9.02) | 47.1 (11.98) | 112 (55) |  |
| FF 100 ug DPI | 0 | 204(74) | 61.8 (8.28) | 48.4 (12.17) | 114 (56) |  |
| Placebo DPI | 0 | 205(74) | 61.9 (8.14) | 48.3 (12.71) | 108 (53) |  |
| **Maltais et al 2020**  **(NCT01957150)** |  |  |  |  |  |  |
| FF/VIn 100/25ug | 12 | 141(50) | 64.4 (9.0) | 58.9 (5.9) | NA | 36 |
| VIn 25ug | 7 | 142(51) | 66.0 (8.2) | 59.5 (6.1) | NA |  |
| **Ferguson et al 2008**  **(SCO40043)** |  |  |  |  |  |  |
| FSC 250/50ug | 3 | 394(58) | 64.9 (9.0) | 39.8 (13.9) | 40 | 12 |
| Salmeterol 50ug | 2 | 388(52) | 65.0 (9.1) | 40.6 (15.4) | 38 |  |
| **Ferguson et al 2017**  **(NCT02157935)** |  |  |  |  |  |  |
| Budesonide/formoterol pMDI 320/9 µg BID | 8 | 606(58.6) | 63.1 (8.65) | 48.54 (12.71) | 279 (46.0) | 6 |
| Formoterol DPI 9 µg BID | 2 | 613(56.0) | 63.9 (8.67) | 48.87 (12.70) | 283 (46.2) |  |
| **Ferguson et al 2018 (KRONOS)**  **(NCT02497001)** |  |  |  |  |  |  |
| BGF MDI 320/18/9·6 µg | 1 | 639(72·0%) | 64·9 (7·8) | 50·2 (14·3) | 256 (40·1%) | 6 |
| GFF MDI 18/9.6 μg | 2 | 625(68·8%) | 65·1 (7·7) | 50·2 (13·8) | 257 (41·1%) |  |
| BFF MDI 320/9.6 μg | 0 | 314(71·3%) | 65·2 (7·2) | 50·0 (14·0) | 115 (36.6%) |  |
| BUD/FORM DPI 400/12ug | 1 | 318 (74·2%) | 65·9 (7·7) | 50.7 (13.8) | 122 (38·4%) |  |
| **Ferguson et al 2018 (TELOS)**  **(NCT02766608)** |  |  |  |  |  |  |
| BFF MDI 320/10 µg | 3 | 655 (61.4) | 64.2±7.7 | 52.92±13.49 | 346 (52.8) | 6 |
| BFF MDI 160/10 µg | 1 | 637 (59.2) | 64.3±7.6 | 53.07±12.82 | 340 (53.4) |  |
| FF MDI 10 µg | 2 | 644(59.5) | 64.1±8.0 | 52.61±12.70 | 348 (54.0) |  |
| BD MDI 320 µg | 1 | 206(60.7) | 64.2±7.4 | 53.40±13.30 | 112 (54.4) |  |
| BUD/FORM DPI 400/12ug | 3 | 219(64.4) | 65.3±7.0 | 53.04±12.79 | 108 (49.3) |  |
| **Magnussen et al 2014(WISDOM)**  **(NCT00975195)** |  |  |  |  |  |  |
| fluticasone/ salmeterol 500/50ug BID tiotropium 18 μg QD | 6 | 1243(81.5) | 63.6±8.6 | 34.2±11.2 | 34.8 | 12 |
| salmeterol 50ug BID tiotropium 18 μg QD | 8 | 1242(83.4) | 64.0±8.4 | 34.3±10.8 | 32.1 |  |
| **Covelli et al 2016**  **(NCT01627327)** |  |  |  |  |  |  |
| FF/VI 100/25 ug | 1 | 310（62） | 62.9 (8.1) | 49.4 (10.5) | 52 | 3 |
| TIO 18 ug | 0 | 313（67) | 62.3 (8.0) | 49.7 (11.1) | 51 |  |
| **Wedzicha et al 2008(INSPIRE)**  **(NCT 00361959)** |  |  |  |  |  |  |
| SFC 50/500 mg bid | 4 | 658(81) | 64 | 39.1 | 38 | 24 |
| Tiotropium 18 mg qd | 1 | 665(84) | 65 | 39.4 | 38 |  |
| **Wedzicha et al 2016 (FLAME)**  **(NCT0178232)** |  |  |  |  |  |  |
| salmeterol (50 ug)/fluticasone (500 ug) bid | 10 | 1682(74.8) | 64.5±7.7 | 44.1±9.4 | 669 (39.8) | 12 |
| indacaterol (110 ug) / glycopyrronium (50 ug) qd | 10 | 1680(77.3) | 64.6±7.9 | 44.0±9.5 | 664 (39.5) |  |
| **Pepin et al 2014**  **(NCT01395888)** |  |  |  |  |  |  |
| FF/VI, 100/25 m g | 1 | 127(85) | 66.7 (7.20) | 45.6 (14.49) | 59 (46) | 3 |
| TIO 18, m g | 0 | 130(86) | 67.7 (7.34) | 47.4 (13.80) | 58 (45) |  |
| **Ohar et al 2014**  **(NCT01110200)** |  |  |  |  |  |  |
| FP/SAL 250/50 μg | 2 | 314(55) | 63.1 (9.15) | 38.5 (14.82) | NA | 6 |
| SAL 50 μg | 1 | 325(54) | 62.7 (9.30) | 41.2 (16.85) | NA |  |
| **Zheng et al 2015**  **(NCT01376245)** |  |  |  |  |  |  |
| FF/VI 200/25 | 0 | 160(91) | 62.7 (8.65) | 48.2 (13.63) | 50 (31) | 6 |
| FF/VI 100/25 | 0 | 161(93) | 65.1 (9.19) | 49.6 (13.19) | 52 (32) |  |
| FF/VI 50/25 | 0 | 160(90) | 65.2 (8.41) | 47.5 (14.21) | 46 (29) |  |
| Placebo | 3 | 162(90) | 64.7 (8.78) | 48.6 | 56 (35) |  |
| **Vestbo et al 2016 (SUMMIT)**  **(NCT01313676)** |  |  |  |  |  |  |
| FF/VI 100/25 ug | 37 | 4140(76) | 65 (8) | 59·7 (6·1) | 1868 (45%) | 36 |
| VI 25 ug | 30 | 4140(74) | 65 (8) | 59·7 (6·1) | 1929 (47%) |  |
| FF 100 ug | 35 | 4157(74) | 65 (8) | 59·6 (6·1) | 1945 (47%) |  |
| Placebo | 23 | 4131(75) | 65 (8) | 59·7 (6·1) | 1936 (47%) |  |
| **Vestbo et al 2017 (TRINITY)**  **(NCT01911364)** |  |  |  |  |  |  |
| BDP/FF/GB | 5 | 1077 (77%) | 63·4 (8·7) | 36.60% | 517 (48%) | 12 |
| Tiotropium | 2 | 1076(77%) | 63·3 (8·4) | 36.60% | 503 (47%) |  |
| open triple | 1 | 537 (74%) | 62·6 (8·9) | 36·7% | 266 (50%) |  |
| **Beeh et al 2016 (ENERGITO)**  **(NCT01969721)** |  |  |  |  |  |  |
| Salmeterol + fluticasone 50ug/500ug BD | 0 | 219(64.6) | 63.6 (7.6) | 56.436 (11.812) | 102 (44.5) | 6 |
| Salmeterol + fluticasone 50/250 µg | 0 | 212(64.6) | 63.6 (7.6) | 56.436 (11.812) | 102 (44.5) |  |
| Tiotropium +olodaterol 5/5 µg | 0 | 221(64.6) | 63.6 (7.6) | 56.436 (11.812) | 102 (44.5) |  |
| Tiotropium +olodaterol 2.5/5 µg | 2 | 215(64.6) | 63.6 (7.6) | 56.436 (11.812) | 102 (44.5) |  |
| **Chapman et al 2018 (SUNSET)**  **(NCT02603393)** |  |  |  |  |  |  |
| Tiotropium /salmeterol/fluticasone | 0 | 526(69.4) | 65.2 ± 7.62 | 57.0 ± 10.30 | NA | 6 |
| Indacaterol/glycopyrronium | 1 | 527(71.7) | 65.4 ± 7.99 | 56.2 ± 9.66 | NA |  |
| **Huang et al 2019**  **(NCT01415518)** |  |  |  |  |  |  |
| BUD/FORM 160/4.5µg + I 20µg/T 100mg bid | 0 | 290(87.6) | 63.8 (8.8) | NA | 69 (23.8) | 3 |
| I 20µg/T 100mg bid | 1 | 291(85.3) | 64.4 (8.8) | NA | 66 (22.6) |  |
| **Rabe et al 2020 (ETHOS)**  **(NCT02465567)** |  |  |  |  |  |  |
| Budesonide/formoterol/glycopyrrolate 320/9.6 µg/18 μgMDI | 19 | 2137(59.0) | 64.6±7.6 | 43.6±10.3 | 910 (42.6) | 12 |
| Budesonide/formoterol/glycopyrrolate 160/9.6 µg/18 μgMDI | 17 | 2121(61.2) | 64.6±7.6 | 43.1±10.4 | 865 (40.8) |  |
| formoterol/glycopyrrolate 9.6 µg/18 μg MDI | 10 | 2120(58.7) | 64.8±7.6 | 43.5±10.2 | 856 (40.4) |  |
| Budesonide/formoterol 320/9.6 µg MDI | 19 | 2131(60.0) | 64.6±7.6 | 43.4±10.4 | 864 (40.5) |  |
| **Dransfield et al 2013**  **(NCT01009463, NCT01017952)** |  |  |  |  |  |  |
| Study 1 |  |  |  |  |  | 12 |
| FF/VI 200 μg/25 μg | 3 | 402(61.9) | 63·8 (9·3) | 45·1% (13·8) | NA |  |
| FF/VI 100/25ug | 0 | 403(57.3) | 63·6 (9·1) | 45·7% (12·9) | NA |  |
| FF/VI 50 μg/25 μg | 1 | 408(60) | 63·6 (9·1) | 45·6% (13·7) | NA |  |
| VI 25 μg | 0 | 409(58.4) | 63.6 (9.4) | 44.3% (13.2) | NA |  |
| Study 2 |  |  |  |  |  |  |
| FF/VI 200 μg/25 μg | 1 | 409(53.3) | 63·5 (8·8) | 45.3% (13.1) | NA |  |
| FF/VI 100/25ug | 3 | 403(55.1) | 64.0 (9.3) | 46·4% (13·9) | NA |  |
| FF/VI 50 μg/25 μg | 3 | 412(56.1) | 63·7 (9·6) | 45·2% (13·5) | NA |  |
| VI 25 μg | 2 | 409(57.5) | 63·6 (9·3) | 46.1% (12.8) | NA |  |
| **Ichinose et al 2019**  **(NCT03262012)** |  |  |  |  |  |  |
| BGF MDI 320/18/9.6 ug bid | 1 | 139(93.5%) | 69.4 (7.1) | NA | NA | 6 |
| GFF MDI 18/9.6 ug bid | 0 | 138(91.3%) | 69.0 (6.1) | NA | NA |  |
| BFF MDI 320/9.6 μg bid | 3 | 70(97.1%) | 69.7 (6.1) | NA | NA |  |
| BUD/FORM DPI 400/12 ug bid | 1 | 69(98.6%) | 70.1 (6.8) | NA | NA |  |
| **Scanlon et al 2004** |  |  |  |  |  |  |
| triamcinolone acetonide, 600 mcg bid | 14 | 201( 53.2) | 55.7 (6.9) | 70.5 (12.6) | 88.1 | 36 |
| placebo bid | 21 | 211(51.7) | 57.1 (6.5) | 68.1 ( 13.1) | 85.3 |  |
| **Calverley et al 2007 (TORCH)**  **(NCT00268216)** |  |  |  |  |  |  |
| Fluticasone/ salmeterol 500/50ug | 97 | 1533(75) | 65.0±8.3 | 44.3±12.3 | 660 (43) | 36 |
| Fluticasone 500ug | 84 | 1534(75) | 65.0±8.4 | 44.1±12.3 | 661 (43) |  |
| SAL 50 μg | 78 | 1521(76) | 65.1±8.2 | 43.6±12.6 | 651 (43) |  |
| Placebo | 78 | 1524(76) | 65.0±8.2 | 44.1±12.3 | 658 (43) |  |
| **Calverley et al 2008** |  |  |  |  |  |  |
| MF-DPI 800 ug QD | 3 | 308(69) | 65.3 | 47 | NA | 12 |
| MF-DPI 400 ug BID | 3 | 308(67) | 65 | 46 | NA |  |
| Placebo | 3 | 295(69) | 65 | 47 | NA |  |
| **Burge et al 2000 (ISOLDE)** |  |  |  |  |  |  |
| Fluticasone | 9 | 376(75) | 63.7 (7.1) | 50.3% (14.9% ) | 137(36.4) | 36 |
| Placebo | 17 | 375(74.1) | 63.8 (7.1) | 50.0% (14.9%) | 147(39.2) |  |
| **Pauwels et al 1999(EUROSCOP)** |  |  |  |  |  |  |
| BUDESONIDE 400 µg | 8 | 634(73.5) | 52.5±7.5 | 76.8±12.4 | NA | 36 |
| PLACEBO | 3 | 643(72.2) | 52.4±7.7 | 76.9±13.2 | NA |  |
| **Bansal et al 2021**  **(NCT03474081)** |  |  |  |  |  |  |
| FF/UMEC/VI 100/62.5/25 ug qd | 1 | 400(69) | 66.2 (8.08) | 49.8 (14.0) | 189 (47) | 3 |
| TIO mcg 1d | 0 | 400(67) | 66.1 (7.78) | 50.2 (14.2) | 192 (48) |  |
| **Lee et al 2016**  **(NCT01397890)** |  |  |  |  |  |  |
| Tiotropium (18 μg od) + budesonide/formoterol (160/4.5 μg, bid) | 1 | 287(97.2) | 66.6 (8.0) | 35.8 (11.3) | NA | 3 |
| Tiotropium alone 18 μg od | 0 | 290(94.1) | 66.9 (8.5) | 37.0 (10.6) | NA |  |
| **Bhatt et al 2017**  **(NCT01336608)** |  |  |  |  |  |  |
| FF/VI 100/25 Ug QD | 3 | 135(77) | 68.5 (8.0) | 51.1 (13.48) | 49 (36) | 6 |
| VI 25 ug qd | 1 | 154(77) | 68.7 (7.7) | 48.8 (12.90) | 57 (37) |  |
| placebo | 0 | 141(84) | 68.2 (8.1) | 50.6 (13.65) | 54 (38) |  |
| **Siler et al 2017**  **(NCT02105974)** |  |  |  |  |  |  |
| FF/VI 100/25 µg | 0 | 806(75) | 65.3 (8.58) | 50.3 (10.33) | 367 (46) | 3 |
| VI 25 µg | 3 | 814(77) | 65.4 (9.02) | 50.5 (10.33) | 363 (45) |  |
| **Welte et al 2009**  **(NCT00496470)** |  |  |  |  |  |  |
| BUD/FORM + TIO | 0 | 329 (76) | 62.4 (40–85) | 38.1 (8.7) | NA | 3 |
| PBO + TIO | 1 | 331 (74) | 62.5 (41–82) | 37.7 (8.5) | NA |  |
