# Supplementary material for: Effect of fracture risk in inhaled corticosteroids in patients with chronic obstructive pulmonary disease: a systematic review and meta-analysis
Source: BMC Pulm Med. 2023 Aug 17;23:304. doi: 10.1186/s12890-023-02602-5 (PMC10436625; doi:10.1186/s12890-023-02602-5)
Supplement: Supplementary file 3 — Additional file 3: Table S3. GRADE summary of findings. [file 12890_2023_2602_MOESM3_ESM.docx]

TABLE S3: GRADE summary of findings

| **Question: ICSs versus Controls for fracture risk Bibliography:** . ICSs versus Control for fracture. Cochrane Database of Systematic Reviews [Year], Issue [Issue]. | | | | | | | | | | | |
| --- | --- | --- | --- | --- | --- | --- | --- | --- | --- | --- | --- |
| **Quality assessment** | | | | | | | **Summary of Findings** | | | | |
| **Participants (studies) Follow up** | **Risk of bias** | **Inconsistency** | **Indirectness** | **Imprecision** | **Publication bias** | **Overall quality of evidence** | **Study event rates (%)** | | **Relative effect** (95% CI) | **Anticipated absolute effects** | |
|  |  |  |  |  |  |  | **With Control** | **With ICSs versus Control for fracture1** |  | **Risk with Control** | **Risk difference with ICSs versus Control for fracture1** (95% CI) |
| **ICSs versus Control for fracture risk** | | | | | | | | | | | |
| 87594 (44 studies) | serious^1^ | no serious inconsistency | no serious indirectness | no serious imprecision | undetected | ⊕⊕⊕⊝ **MODERATE**^1^ due to risk of bias | 351/34964  (1%) | 532/52630  (1%) | **RR 1.19**  (1.04 to 1.37) | **Study population** | |
|  |  |  |  |  |  |  |  |  |  | **10 per 1000** | **2 more per 1000** (from 0 more to 4 more) |
|  |  |  |  |  |  |  |  |  |  | **Moderate** | |
|  |  |  |  |  |  |  |  |  |  | **3 per 1000** | **1 more per 1000** (from 0 more to 1 more) |
| **ICSs versus Control for fracture risk - < 12 months** | | | | | | | | | | | |
| 19840 (21 studies) | serious^1^ | no serious inconsistency | no serious indirectness | serious^2^ | undetected | ⊕⊕⊝⊝ **LOW**^1,2^ due to risk of bias, imprecision | 29/8354  (0.3%) | 48/11486  (0.4%) | **RR 1.2**  (0.79 to 1.81) | **Study population** | |
|  |  |  |  |  |  |  |  |  |  | **3 per 1000** | **1 more per 1000** (from 1 fewer to 3 more) |
|  |  |  |  |  |  |  |  |  |  | **Moderate** | |
|  |  |  |  |  |  |  |  |  |  | **3 per 1000** | **1 more per 1000** (from 1 fewer to 2 more) |
| **ICSs versus Control for fracture risk - ≥ 12 months** | | | | | | | | | | | |
| 67387 (23 studies) | no serious risk of bias | no serious inconsistency | no serious indirectness | no serious imprecision | undetected | ⊕⊕⊕⊕ **HIGH** | 326/26417  (1.2%) | 489/40970  (1.2%) | **RR 1.19**  (1.04 to 1.38) | **Study population** | |
|  |  |  |  |  |  |  |  |  |  | **12 per 1000** | **2 more per 1000** (from 0 more to 5 more) |
|  |  |  |  |  |  |  |  |  |  | **Moderate** | |
|  |  |  |  |  |  |  |  |  |  | **6 per 1000** | **1 more per 1000** (from 0 more to 2 more) |
| **ICSs versus Control for fracture risk - Triamcinolone** | | | | | | | | | | | |
| 412 (1 study) | serious^3^ | no serious inconsistency | no serious indirectness | very serious^2,4^ | undetected | ⊕⊝⊝⊝ **VERY LOW**^2,3,4^ due to risk of bias, imprecision | 21/211  (10%) | 14/201  (7%) | **RR 0.7**  (0.37 to 1.34) | **Study population** | |
|  |  |  |  |  |  |  |  |  |  | **100 per 1000** | **30 fewer per 1000** (from 63 fewer to 34 more) |
|  |  |  |  |  |  |  |  |  |  | **Moderate** | |
|  |  |  |  |  |  |  |  |  |  | **100 per 1000** | **30 fewer per 1000** (from 63 fewer to 34 more) |
| **ICSs versus Control for fracture risk - Mometasone** | | | | | | | | | | | |
| 3162 (3 studies) | serious^3^ | no serious inconsistency | no serious indirectness | serious^2^ | undetected | ⊕⊕⊝⊝ **LOW**^2,3^ due to risk of bias, imprecision | 10/1195  (0.8%) | 16/1967  (0.8%) | **RR 0.95**  (0.43 to 2.1) | **Study population** | |
|  |  |  |  |  |  |  |  |  |  | **8 per 1000** | **0 fewer per 1000** (from 5 fewer to 9 more) |
|  |  |  |  |  |  |  |  |  |  | **Moderate** | |
|  |  |  |  |  |  |  |  |  |  | **10 per 1000** | **1 fewer per 1000** (from 6 fewer to 11 more) |
| **ICSs versus Control for fracture risk - Beclometasone** | | | | | | | | | | | |
| 4222 (2 studies) | no serious risk of bias | no serious inconsistency | no serious indirectness | serious^2^ | undetected | ⊕⊕⊕⊝ **MODERATE**^2^ due to imprecision | 5/1844  (0.3%) | 8/2378  (0.3%) | **RR 1.26**  (0.4 to 3.99) | **Study population** | |
|  |  |  |  |  |  |  |  |  |  | **3 per 1000** | **1 more per 1000** (from 2 fewer to 8 more) |
|  |  |  |  |  |  |  |  |  |  | **Moderate** | |
|  |  |  |  |  |  |  |  |  |  | **3 per 1000** | **1 more per 1000** (from 2 fewer to 9 more) |
| **ICSs versus Control for fracture risk - Budesonide** | | | | | | | | | | | |
| 20874 (12 studies) | no serious risk of bias | no serious inconsistency | no serious indirectness | no serious imprecision | undetected | ⊕⊕⊕⊕ **HIGH**^5^ due to dose-response gradient | 26/6857  (0.4%) | 93/14017  (0.7%) | **RR 1.64**  (1.07 to 2.51) | **Study population** | |
|  |  |  |  |  |  |  |  |  |  | **4 per 1000** | **2 more per 1000** (from 0 more to 6 more) |
|  |  |  |  |  |  |  |  |  |  | **Moderate** | |
|  |  |  |  |  |  |  |  |  |  | **3 per 1000** | **2 more per 1000** (from 0 more to 5 more) |
| **ICSs versus Control for fracture risk - Budesonide 160 ug bid** | | | | | | | | | | | |
| 8256 (6 studies) | serious^1^ | no serious inconsistency | no serious indirectness | serious^2^ | undetected | ⊕⊕⊝⊝ **LOW**^1,2^ due to risk of bias, imprecision | 17/4232  (0.4%) | 20/4024  (0.5%) | **RR 1.17**  (0.62 to 2.18) | **Study population** | |
|  |  |  |  |  |  |  |  |  |  | **4 per 1000** | **1 more per 1000** (from 2 fewer to 5 more) |
|  |  |  |  |  |  |  |  |  |  | **Moderate** | |
|  |  |  |  |  |  |  |  |  |  | **3 per 1000** | **1 more per 1000** (from 1 fewer to 4 more) |
| **ICSs versus Control for fracture risk - Budesonide 320 ug bid** | | | | | | | | | | | |
| 14342 (9 studies) | no serious risk of bias | no serious inconsistency | no serious indirectness | no serious imprecision | undetected | ⊕⊕⊕⊕ **HIGH** | 22/5595  (0.4%) | 60/8747  (0.7%) | **RR 1.66**  (1.03 to 2.7) | **Study population** | |
|  |  |  |  |  |  |  |  |  |  | **4 per 1000** | **3 more per 1000** (from 0 more to 7 more) |
|  |  |  |  |  |  |  |  |  |  | **Moderate** | |
|  |  |  |  |  |  |  |  |  |  | **3 per 1000** | **2 more per 1000** (from 0 more to 5 more) |
| **ICSs versus Control for fracture risk - Fluticasone** | | | | | | | | | | | |
| 58924 (26 studies) | no serious risk of bias | no serious inconsistency | no serious indirectness | no serious imprecision | undetected | ⊕⊕⊕⊕ **HIGH** | 289/24857  (1.2%) | 401/34067  (1.2%) | **RR 1.18**  (1.02 to 1.38) | **Study population** | |
|  |  |  |  |  |  |  |  |  |  | **12 per 1000** | **2 more per 1000** (from 0 more to 4 more) |
|  |  |  |  |  |  |  |  |  |  | **Moderate** | |
|  |  |  |  |  |  |  |  |  |  | **4 per 1000** | **1 more per 1000** (from 0 more to 2 more) |
| **ICSs versus Control for fracture risk - Fluticasone 50 ug qd** | | | | | | | | | | | |
| 2578 (3 studies) | no serious risk of bias | no serious inconsistency | no serious indirectness | serious^2^ | undetected | ⊕⊕⊕⊝ **MODERATE**^2^ due to imprecision | 8/1392  (0.6%) | 4/1186  (0.3%) | **RR 0.66**  (0.21 to 2.05) | **Study population** | |
|  |  |  |  |  |  |  |  |  |  | **6 per 1000** | **2 fewer per 1000** (from 5 fewer to 6 more) |
|  |  |  |  |  |  |  |  |  |  | **Moderate** | |
|  |  |  |  |  |  |  |  |  |  | **7 per 1000** | **2 fewer per 1000** (from 6 fewer to 7 more) |
| **ICSs versus Control for fracture risk - Fluticasone 100 ug qd** | | | | | | | | | | | |
| 34522 (12 studies) | serious^1^ | no serious inconsistency | no serious indirectness | no serious imprecision | undetected | ⊕⊕⊕⊝ **MODERATE**^1^ due to risk of bias | 82/14235  (0.6%) | 160/20287  (0.8%) | **RR 1.37**  (1.04 to 1.8) | **Study population** | |
|  |  |  |  |  |  |  |  |  |  | **6 per 1000** | **2 more per 1000** (from 0 more to 5 more) |
|  |  |  |  |  |  |  |  |  |  | **Moderate** | |
|  |  |  |  |  |  |  |  |  |  | **4 per 1000** | **1 more per 1000** (from 0 more to 3 more) |
| **ICSs versus Control for fracture risk - Fluticasone 200 ug qd** | | | | | | | | | | | |
| 2767 (3 studies) | no serious risk of bias | no serious inconsistency | no serious indirectness | serious^2^ | undetected | ⊕⊕⊕⊝ **MODERATE**^2^ due to imprecision | 5/1388  (0.4%) | 6/1379  (0.4%) | **RR 1.18**  (0.4 to 3.5) | **Study population** | |
|  |  |  |  |  |  |  |  |  |  | **4 per 1000** | **1 more per 1000** (from 2 fewer to 9 more) |
|  |  |  |  |  |  |  |  |  |  | **Moderate** | |
|  |  |  |  |  |  |  |  |  |  | **2 per 1000** | **0 more per 1000** (from 1 fewer to 5 more) |
| **ICSs versus Control for fracture risk - Fluticasone 250 ug bid** | | | | | | | | | | | |
| 4044 (5 studies) | serious^3^ | no serious inconsistency | no serious indirectness | serious^2^ | undetected | ⊕⊕⊝⊝ **LOW**^2,3^ due to risk of bias, imprecision | 7/2142  (0.3%) | 11/1902  (0.6%) | **RR 1.72**  (0.68 to 4.34) | **Study population** | |
|  |  |  |  |  |  |  |  |  |  | **3 per 1000** | **2 more per 1000** (from 1 fewer to 11 more) |
|  |  |  |  |  |  |  |  |  |  | **Moderate** | |
|  |  |  |  |  |  |  |  |  |  | **3 per 1000** | **2 more per 1000** (from 1 fewer to 10 more) |
| **ICSs versus Control for fracture risk - Fluticasone 500 ug bid** | | | | | | | | | | | |
| 18898 (11 studies) | serious^1^ | no serious inconsistency | no serious indirectness | serious^2^ | undetected | ⊕⊕⊝⊝ **LOW**^1,2^ due to risk of bias, imprecision | 204/9552  (2.1%) | 225/9346  (2.4%) | **RR 1.1**  (0.92 to 1.32) | **Study population** | |
|  |  |  |  |  |  |  |  |  |  | **21 per 1000** | **2 more per 1000** (from 2 fewer to 7 more) |
|  |  |  |  |  |  |  |  |  |  | **Moderate** | |
|  |  |  |  |  |  |  |  |  |  | **5 per 1000** | **1 more per 1000** (from 0 fewer to 2 more) |
| **ICSs versus Control for fracture risk - Mean age,yeas < 65** | | | | | | | | | | | |
| 45519 (30 studies) | serious^3^ | no serious inconsistency | no serious indirectness | serious^2^ | undetected | ⊕⊕⊝⊝ **LOW**^2,3^ due to risk of bias, imprecision | 115/17519  (0.7%) | 183/28000  (0.7%) | **RR 1.08**  (0.85 to 1.37) | **Study population** | |
|  |  |  |  |  |  |  |  |  |  | **7 per 1000** | **1 more per 1000** (from 1 fewer to 2 more) |
|  |  |  |  |  |  |  |  |  |  | **Moderate** | |
|  |  |  |  |  |  |  |  |  |  | **4 per 1000** | **0 more per 1000** (from 1 fewer to 1 more) |
| **ICSs versus Control for fracture risk - Mean age,years ≥ 65** | | | | | | | | | | | |
| 42075 (14 studies) | serious^3^ | no serious inconsistency | no serious indirectness | no serious imprecision | undetected | ⊕⊕⊕⊝ **MODERATE**^3^ due to risk of bias | 236/17445  (1.4%) | 349/24630  (1.4%) | **RR 1.26**  (1.07 to 1.48) | **Study population** | |
|  |  |  |  |  |  |  |  |  |  | **14 per 1000** | **4 more per 1000** (from 1 more to 6 more) |
|  |  |  |  |  |  |  |  |  |  | **Moderate** | |
|  |  |  |  |  |  |  |  |  |  | **3 per 1000** | **1 more per 1000** (from 0 more to 1 more) |
| **ICSs versus Control for fracture risk - GOLD 2** | | | | | | | | | | | |
| 28500 (12 studies) | serious^3^ | no serious inconsistency | no serious indirectness | serious^2^ | undetected | ⊕⊕⊝⊝ **LOW**^2,3^ due to risk of bias, imprecision | 96/13475  (0.7%) | 125/15025  (0.8%) | **RR 1.26**  (0.97 to 1.63) | **Study population** | |
|  |  |  |  |  |  |  |  |  |  | **7 per 1000** | **2 more per 1000** (from 0 fewer to 4 more) |
|  |  |  |  |  |  |  |  |  |  | **Moderate** | |
|  |  |  |  |  |  |  |  |  |  | **4 per 1000** | **1 more per 1000** (from 0 fewer to 3 more) |
| **ICSs versus Control for fracture - GOLD 3** | | | | | | | | | | | |
| 56585 (28 studies) | no serious risk of bias | no serious inconsistency | no serious indirectness | no serious imprecision | undetected | ⊕⊕⊕⊕ **HIGH** | 247/20464  (1.2%) | 394/36121  (1.1%) | **RR 1.18**  (1 to 1.38) | **Study population** | |
|  |  |  |  |  |  |  |  |  |  | **12 per 1000** | **2 more per 1000** (from 0 more to 5 more) |
|  |  |  |  |  |  |  |  |  |  | **Moderate** | |
|  |  |  |  |  |  |  |  |  |  | **3 per 1000** | **1 more per 1000** (from 0 more to 1 more) |
| **Triple Therapy versus Control for fracture** | | | | | | | | | | | |
| 24887 (13 studies) | no serious risk of bias | no serious inconsistency | no serious indirectness | no serious imprecision | undetected | ⊕⊕⊕⊕ **HIGH** | 39/10053  (0.4%) | 98/14834  (0.7%) | **RR 1.49**  (1.03 to 2.17) | **Study population** | |
|  |  |  |  |  |  |  |  |  |  | **4 per 1000** | **2 more per 1000** (from 0 more to 5 more) |
|  |  |  |  |  |  |  |  |  |  | **Moderate** | |
|  |  |  |  |  |  |  |  |  |  | **3 per 1000** | **1 more per 1000** (from 0 more to 4 more) |
| **ICS/LABA versus Control for fracture** | | | | | | | | | | | |
| 56250 (31 studies) | no serious risk of bias | no serious inconsistency | no serious indirectness | no serious imprecision | undetected | ⊕⊕⊕⊕ **HIGH** | 292/28106  (1%) | 274/28144  (1%) | **RR 1.3**  (1.1 to 1.53) | **Study population** | |
|  |  |  |  |  |  |  |  |  |  | **10 per 1000** | **3 more per 1000** (from 1 more to 6 more) |
|  |  |  |  |  |  |  |  |  |  | **Moderate** | |
|  |  |  |  |  |  |  |  |  |  | **3 per 1000** | **1 more per 1000** (from 0 more to 2 more) |
| **ICS vs Placebo for fracture** | | | | | | | | | | | |
| 17557 (12 studies) | no serious risk of bias | no serious inconsistency | no serious indirectness | serious^2^ | undetected | ⊕⊕⊕⊝ **MODERATE**^2^ due to imprecision | 149/8520  (1.7%) | 164/9037  (1.8%) | **RR 1.07**  (0.86 to 1.33) | **Study population** | |
|  |  |  |  |  |  |  |  |  |  | **17 per 1000** | **1 more per 1000** (from 2 fewer to 6 more) |
|  |  |  |  |  |  |  |  |  |  | **Moderate** | |
|  |  |  |  |  |  |  |  |  |  | **5 per 1000** | **0 more per 1000** (from 1 fewer to 2 more) |
| **Triple therapy versus LAMA/LABA for fracture** | | | | | | | | | | | |
| 19578 (8 studies) | no serious risk of bias | no serious inconsistency | no serious indirectness | no serious imprecision | undetected | ⊕⊕⊕⊕ **HIGH** | 35/7664  (0.5%) | 90/11914  (0.8%) | **RR 1.51**  (1.01 to 2.25) | **Study population** | |
|  |  |  |  |  |  |  |  |  |  | **5 per 1000** | **2 more per 1000** (from 0 more to 6 more) |
|  |  |  |  |  |  |  |  |  |  | **Moderate** | |
|  |  |  |  |  |  |  |  |  |  | **4 per 1000** | **2 more per 1000** (from 0 more to 5 more) |
| **Triple therapy versus LAMA for fracture** | | | | | | | | | | | |
| 5309 (5 studies) | serious^1^ | no serious inconsistency | no serious indirectness | serious^2^ | undetected | ⊕⊕⊝⊝ **LOW**^1,2^ due to risk of bias, imprecision | 4/2389  (0.2%) | 8/2920  (0.3%) | **RR 1.38**  (0.49 to 3.88) | **Study population** | |
|  |  |  |  |  |  |  |  |  |  | **2 per 1000** | **1 more per 1000** (from 1 fewer to 5 more) |
|  |  |  |  |  |  |  |  |  |  | **Moderate** | |
|  |  |  |  |  |  |  |  |  |  | **2 per 1000** | **1 more per 1000** (from 1 fewer to 6 more) |
| **Triple therapy versus LAMA/LABA for fracture - 6 months** | | | | | | | | | | | |
| 2594 (3 studies) | serious^3^ | no serious inconsistency | no serious indirectness | serious^2^ | undetected | ⊕⊕⊝⊝ **LOW**^2,3^ due to risk of bias, imprecision | 3/1290  (0.2%) | 2/1304  (0.2%) | **RR 0.74**  (0.17 to 3.31) | **Study population** | |
|  |  |  |  |  |  |  |  |  |  | **2 per 1000** | **1 fewer per 1000** (from 2 fewer to 5 more) |
|  |  |  |  |  |  |  |  |  |  | **Moderate** | |
|  |  |  |  |  |  |  |  |  |  | **2 per 1000** | **1 fewer per 1000** (from 2 fewer to 5 more) |
| **Triple therapy versus LAMA/LABA for fracture - 12 months** | | | | | | | | | | | |
| 16984 (5 studies) | no serious risk of bias | no serious inconsistency | no serious indirectness | no serious imprecision | undetected | ⊕⊕⊕⊕ **HIGH** | 32/6374  (0.5%) | 88/10610  (0.8%) | **RR 1.59**  (1.05 to 2.41) | **Study population** | |
|  |  |  |  |  |  |  |  |  |  | **5 per 1000** | **3 more per 1000** (from 0 more to 7 more) |
|  |  |  |  |  |  |  |  |  |  | **Moderate** | |
|  |  |  |  |  |  |  |  |  |  | **5 per 1000** | **3 more per 1000** (from 0 more to 7 more) |
| **Triple therapy versus LAMA/LABA for fracture - Mean age,years < 65** | | | | | | | | | | | |
| 10763 (4 studies) | no serious risk of bias | no serious inconsistency | no serious indirectness | serious^2^ | undetected | ⊕⊕⊕⊝ **MODERATE**^2^ due to imprecision | 22/4304  (0.5%) | 45/6459  (0.7%) | **RR 1.29**  (0.76 to 2.2) | **Study population** | |
|  |  |  |  |  |  |  |  |  |  | **5 per 1000** | **1 more per 1000** (from 1 fewer to 6 more) |
|  |  |  |  |  |  |  |  |  |  | **Moderate** | |
|  |  |  |  |  |  |  |  |  |  | **5 per 1000** | **1 more per 1000** (from 1 fewer to 6 more) |
| **Triple therapy versus LAMA/LABA for fracture - Mean age,years ≥ 65** | | | | | | | | | | | |
| 8815 (4 studies) | serious^3^ | no serious inconsistency | no serious indirectness | serious^2^ | undetected | ⊕⊕⊝⊝ **LOW**^2,3^ due to risk of bias, imprecision | 13/3360  (0.4%) | 45/5455  (0.8%) | **RR 1.82**  (0.99 to 3.36) | **Study population** | |
|  |  |  |  |  |  |  |  |  |  | **4 per 1000** | **3 more per 1000** (from 0 fewer to 9 more) |
|  |  |  |  |  |  |  |  |  |  | **Moderate** | |
|  |  |  |  |  |  |  |  |  |  | **3 per 1000** | **2 more per 1000** (from 0 fewer to 7 more) |
| **Triple therapy versus LAMA/LABA for fracture - GOLD 2** | | | | | | | | | | | |
| 2317 (2 studies) | no serious risk of bias | no serious inconsistency | no serious indirectness | serious^2^ | undetected | ⊕⊕⊕⊝ **MODERATE**^2^ due to imprecision | 3/1152  (0.3%) | 1/1165  (0.1%) | **RR 0.42**  (0.06 to 2.86) | **Study population** | |
|  |  |  |  |  |  |  |  |  |  | **3 per 1000** | **2 fewer per 1000** (from 2 fewer to 5 more) |
|  |  |  |  |  |  |  |  |  |  | **Moderate** | |
|  |  |  |  |  |  |  |  |  |  | **3 per 1000** | **2 fewer per 1000** (from 3 fewer to 6 more) |
| **Triple therapy versus LAMA/LABA for fracture - GOLD 3** | | | | | | | | | | | |
| 16616 (4 studies) | no serious risk of bias | no serious inconsistency | no serious indirectness | no serious imprecision | undetected | ⊕⊕⊕⊕ **HIGH** | 31/6200  (0.5%) | 87/10416  (0.8%) | **RR 1.61**  (1.05 to 2.45) | **Study population** | |
|  |  |  |  |  |  |  |  |  |  | **5 per 1000** | **3 more per 1000** (from 0 more to 7 more) |
|  |  |  |  |  |  |  |  |  |  | **Moderate** | |
|  |  |  |  |  |  |  |  |  |  | **5 per 1000** | **3 more per 1000** (from 0 more to 7 more) |
| **ICS/LABA versus LAMA/LABA for fracture** | | | | | | | | | | | |
| 17413 (8 studies) | no serious risk of bias | no serious inconsistency | no serious indirectness | serious^2^ | undetected | ⊕⊕⊕⊝ **MODERATE**^2^ due to imprecision | 36/7710  (0.5%) | 63/9703  (0.6%) | **RR 1.4**  (0.94 to 2.1) | **Study population** | |
|  |  |  |  |  |  |  |  |  |  | **5 per 1000** | **2 more per 1000** (from 0 fewer to 5 more) |
|  |  |  |  |  |  |  |  |  |  | **Moderate** | |
|  |  |  |  |  |  |  |  |  |  | **5 per 1000** | **2 more per 1000** (from 0 fewer to 5 more) |
| **ICS/LABA versus LAMA for fracture** | | | | | | | | | | | |
| 2203 (3 studies) | no serious risk of bias | no serious inconsistency | no serious indirectness | serious^2^ | undetected | ⊕⊕⊕⊝ **MODERATE**^2^ due to imprecision | 1/1108  (0.1%) | 6/1095  (0.5%) | **RR 3.55**  (0.74 to 17.03) | **Study population** | |
|  |  |  |  |  |  |  |  |  |  | **1 per 1000** | **2 more per 1000** (from 0 fewer to 14 more) |
|  |  |  |  |  |  |  |  |  |  | **Moderate** | |
|  |  |  |  |  |  |  |  |  |  | **0 per 1000** | **-** |
| **Triple therapy versus LABA for fracture** | | | | | | | | | | | |
| 29059 (19 studies) | no serious risk of bias | no serious inconsistency | no serious indirectness | no serious imprecision | undetected | ⊕⊕⊕⊕ **HIGH** | 146/12194  (1.2%) | 204/16865  (1.2%) | **RR 1.24**  (1.01 to 1.52) | **Study population** | |
|  |  |  |  |  |  |  |  |  |  | **12 per 1000** | **3 more per 1000** (from 0 more to 6 more) |
|  |  |  |  |  |  |  |  |  |  | **Moderate** | |
|  |  |  |  |  |  |  |  |  |  | **5 per 1000** | **1 more per 1000** (from 0 more to 3 more) |
| **ICS/LABA versus Placebo for fracture** | | | | | | | | | | | |
| 13249 (6 studies) | serious^1^ | no serious inconsistency | no serious indirectness | no serious imprecision | undetected | ⊕⊕⊕⊝ **MODERATE**^1^ due to risk of bias | 105/6371  (1.6%) | 143/6878  (2.1%) | **RR 1.32**  (1.04 to 1.69) | **Study population** | |
|  |  |  |  |  |  |  |  |  |  | **16 per 1000** | **5 more per 1000** (from 1 more to 11 more) |
|  |  |  |  |  |  |  |  |  |  | **Moderate** | |
|  |  |  |  |  |  |  |  |  |  | **5 per 1000** | **2 more per 1000** (from 0 more to 3 more) |
| **ICS/LABA versus LABA for fracture - 3 months** | | | | | | | | | | | |
| 1620 (1 study) | no serious risk of bias | no serious inconsistency | no serious indirectness | very serious^2,4^ | undetected | ⊕⊕⊝⊝ **LOW**^2,4^ due to imprecision | 3/814  (0.4%) | 0/806  (0%) | **RR 0.14**  (0.01 to 2.79) | **Study population** | |
|  |  |  |  |  |  |  |  |  |  | **4 per 1000** | **3 fewer per 1000** (from 4 fewer to 7 more) |
|  |  |  |  |  |  |  |  |  |  | **Moderate** | |
|  |  |  |  |  |  |  |  |  |  | **4 per 1000** | **3 fewer per 1000** (from 4 fewer to 7 more) |
| **ICS/LABA versus LABA for fracture - 6 months** | | | | | | | | | | | |
| 6372 (7 studies) | no serious risk of bias | no serious inconsistency | no serious indirectness | serious^2^ | undetected | ⊕⊕⊕⊝ **MODERATE**^2^ due to imprecision | 9/2385  (0.4%) | 22/3987  (0.6%) | **RR 1.67**  (0.83 to 3.37) | **Study population** | |
|  |  |  |  |  |  |  |  |  |  | **4 per 1000** | **3 more per 1000** (from 1 fewer to 9 more) |
|  |  |  |  |  |  |  |  |  |  | **Moderate** | |
|  |  |  |  |  |  |  |  |  |  | **4 per 1000** | **3 more per 1000** (from 1 fewer to 9 more) |
| **ICS/LABA versus LABA for fracture - 12 months** | | | | | | | | | | | |
| 9450 (8 studies) | serious^3^ | no serious inconsistency | no serious indirectness | serious^2^ | undetected | ⊕⊕⊝⊝ **LOW**^2,3^ due to risk of bias, imprecision | 19/3192  (0.6%) | 36/6258  (0.6%) | **RR 1.07**  (0.62 to 1.83) | **Study population** | |
|  |  |  |  |  |  |  |  |  |  | **6 per 1000** | **0 more per 1000** (from 2 fewer to 5 more) |
|  |  |  |  |  |  |  |  |  |  | **Moderate** | |
|  |  |  |  |  |  |  |  |  |  | **4 per 1000** | **0 more per 1000** (from 2 fewer to 3 more) |
| **ICS/LABA versus LABA for fracture - 36 months** | | | | | | | | | | | |
| 11617 (3 studies) | no serious risk of bias | no serious inconsistency | no serious indirectness | no serious imprecision | undetected | ⊕⊕⊕⊕ **HIGH** | 115/5803  (2%) | 146/5814  (2.5%) | **RR 1.26**  (1 to 1.6) | **Study population** | |
|  |  |  |  |  |  |  |  |  |  | **20 per 1000** | **5 more per 1000** (from 0 more to 12 more) |
|  |  |  |  |  |  |  |  |  |  | **Moderate** | |
|  |  |  |  |  |  |  |  |  |  | **49 per 1000** | **13 more per 1000** (from 0 more to 29 more) |
| **ICS/LABA versus LABA for fracture - Mean age,years < 65** | | | | | | | | | | | |
| 14736 (13 studies) | no serious risk of bias | no serious inconsistency | no serious indirectness | serious^2^ | undetected | ⊕⊕⊕⊝ **MODERATE**^2^ due to imprecision | 27/5020  (0.5%) | 52/9716  (0.5%) | **RR 1.12**  (0.72 to 1.76) | **Study population** | |
|  |  |  |  |  |  |  |  |  |  | **5 per 1000** | **1 more per 1000** (from 2 fewer to 4 more) |
|  |  |  |  |  |  |  |  |  |  | **Moderate** | |
|  |  |  |  |  |  |  |  |  |  | **4 per 1000** | **0 more per 1000** (from 1 fewer to 3 more) |
| **ICS/LABA versus LABA for fracture - Mean age,years ≥ 65** | | | | | | | | | | | |
| 14323 (6 studies) | serious^1^ | no serious inconsistency | no serious indirectness | no serious imprecision | undetected | ⊕⊕⊕⊝ **MODERATE**^1^ due to risk of bias | 119/7174  (1.7%) | 152/7149  (2.1%) | **RR 1.27**  (1.01 to 1.61) | **Study population** | |
|  |  |  |  |  |  |  |  |  |  | **17 per 1000** | **4 more per 1000** (from 0 more to 10 more) |
|  |  |  |  |  |  |  |  |  |  | **Moderate** | |
|  |  |  |  |  |  |  |  |  |  | **7 per 1000** | **2 more per 1000** (from 0 more to 4 more) |
| **ICS/LABA versus LABA for fracture - GOLD 2** | | | | | | | | | | | |
| 12627 (5 studies) | no serious risk of bias | no serious inconsistency | no serious indirectness | serious^3^ | undetected | ⊕⊕⊕⊝ **MODERATE**^3^ due to imprecision | 43/5894  (0.7%) | 59/6733  (0.9%) | **RR 1.29**  (0.87 to 1.9) | **Study population** | |
|  |  |  |  |  |  |  |  |  |  | **7 per 1000** | **2 more per 1000** (from 1 fewer to 7 more) |
|  |  |  |  |  |  |  |  |  |  | **Moderate** | |
|  |  |  |  |  |  |  |  |  |  | **7 per 1000** | **2 more per 1000** (from 1 fewer to 6 more) |
| **ICS/LABA versus LABA for fracture - GOLD 3** | | | | | | | | | | | |
| 15799 (13 studies) | no serious risk of bias | no serious inconsistency | no serious indirectness | no serious imprecision | undetected | ⊕⊕⊕⊕ **HIGH** | 98/6091  (1.6%) | 141/9708  (1.5%) | **RR 1.27**  (0.99 to 1.63) | **Study population** | |
|  |  |  |  |  |  |  |  |  |  | **16 per 1000** | **4 more per 1000** (from 0 fewer to 10 more) |
|  |  |  |  |  |  |  |  |  |  | **Moderate** | |
|  |  |  |  |  |  |  |  |  |  | **4 per 1000** | **1 more per 1000** (from 0 fewer to 3 more) |

^1^ High-risk bias within a group.
^2^ Merging effect over the equivalent line.
^3^ Most of the information comes from medium bias.
^4^ Small sample size.
^5^ dose-dependet.
