# Supplementary figures and images for: Effect of fracture risk in inhaled corticosteroids in patients with chronic obstructive pulmonary disease: a systematic review and meta-analysis
Source: BMC Pulm Med. 2023 Aug 17;23:304. doi: 10.1186/s12890-023-02602-5 (PMC10436625; doi:10.1186/s12890-023-02602-5)

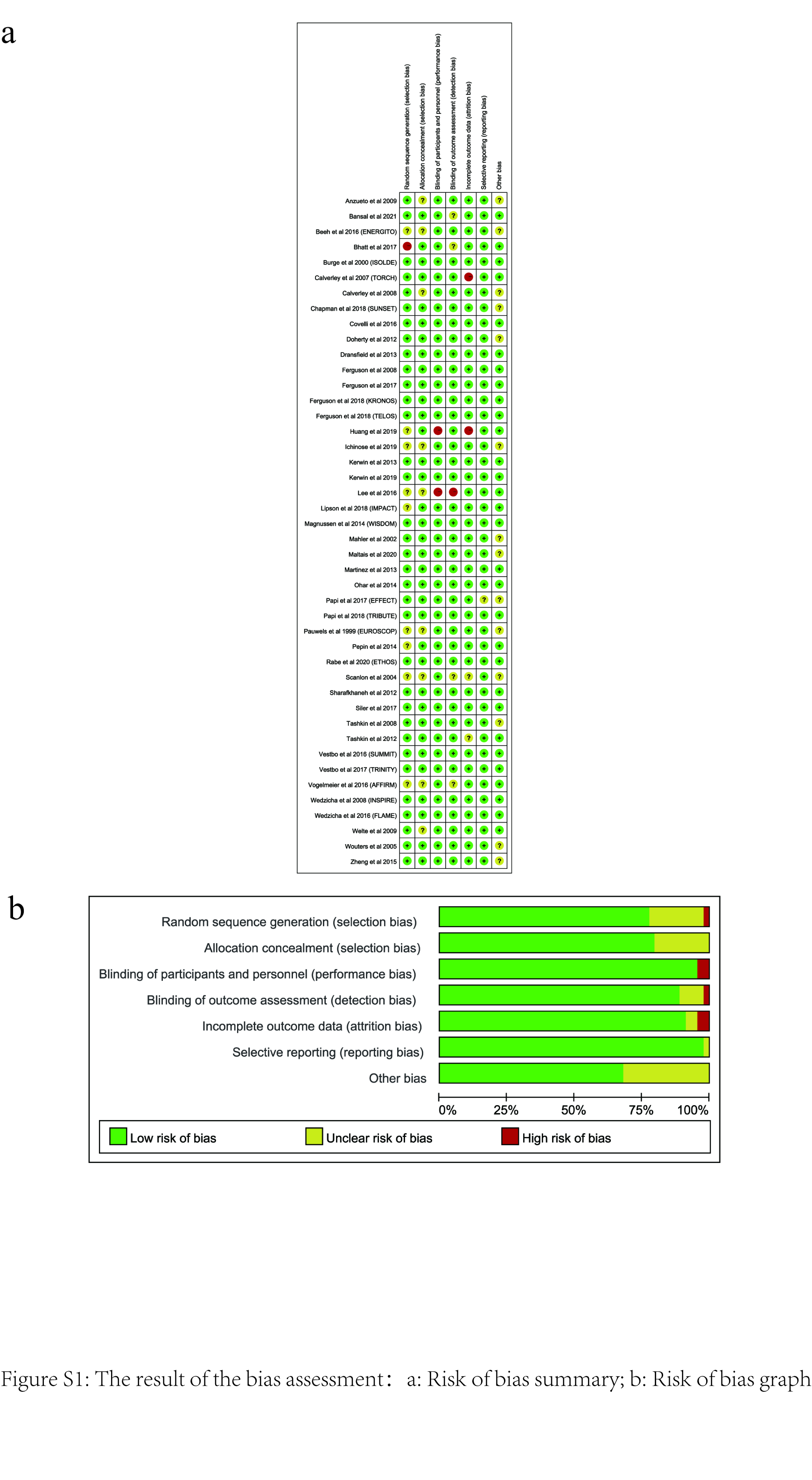

Supplement: Supplementary file 4 — Additional file 4: Figure S1. The result of the bias assessment: a: Risk of bias summary; b: Risk of bias graph. [file 12890_2023_2602_MOESM4_ESM.tif]

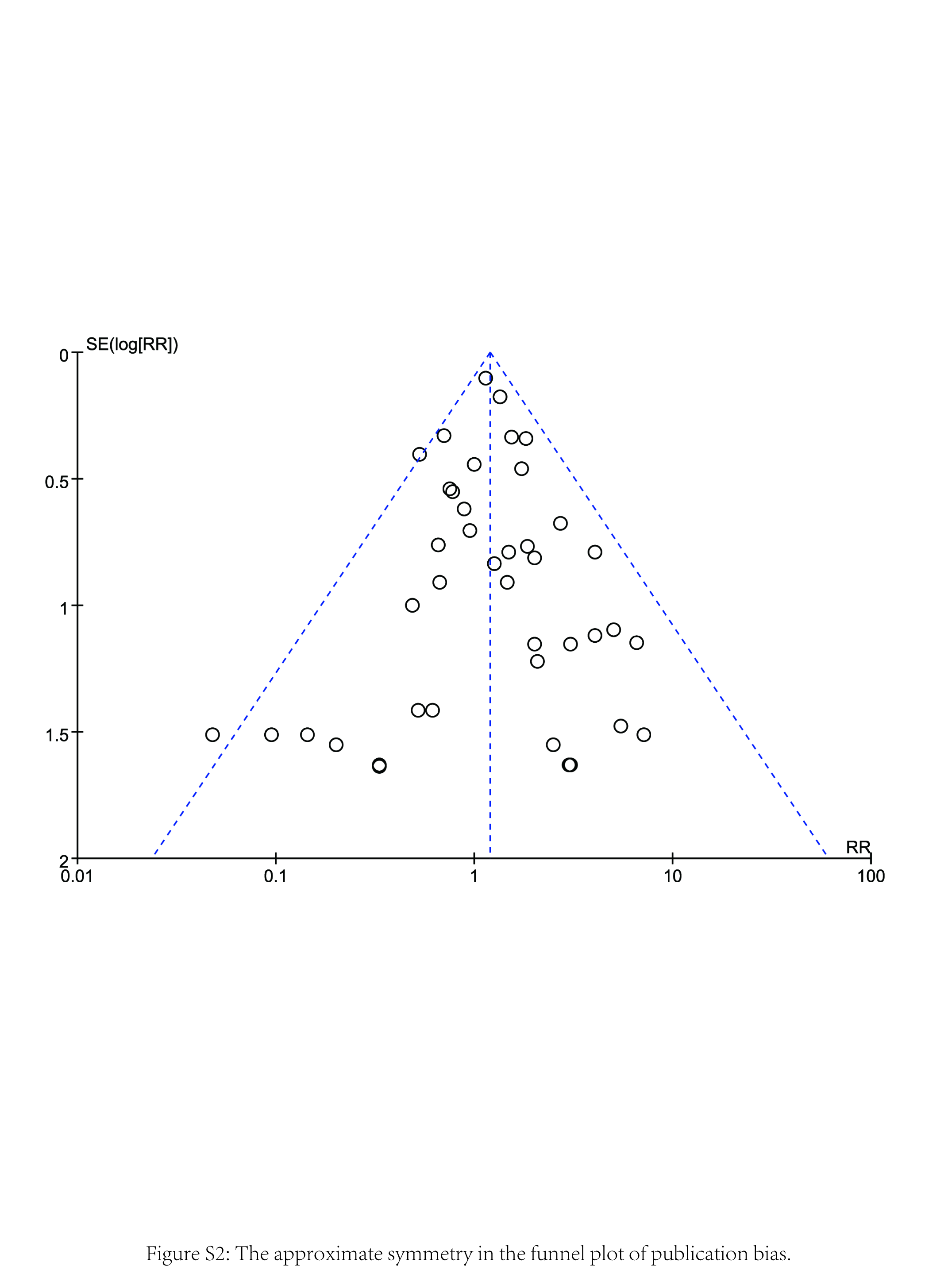

Supplement: Supplementary file 5 — Additional file 5: Figure S2. The approximate symmetry in the funnel plot of publication bias. [file 12890_2023_2602_MOESM5_ESM.tif]

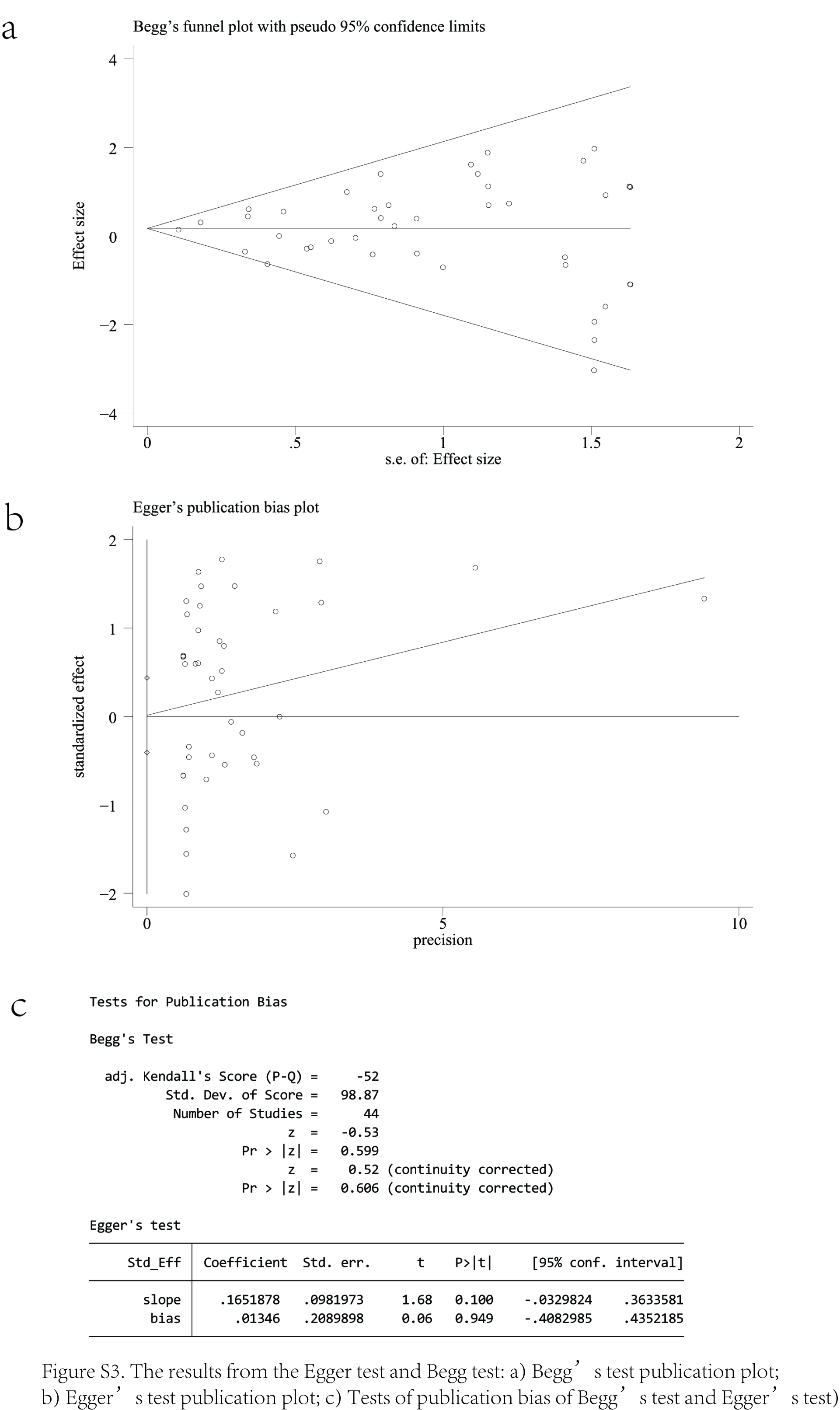

Supplement: Supplementary file 6 — Additional file 6: Figure S3. The results from the Egger test and Begg test: a) Begg’ s test publication plot; b)Egger’s test publication plot; c) Test of publication bias of Begg’ s test and Egger’ s test). [file 12890_2023_2602_MOESM6_ESM.tif]

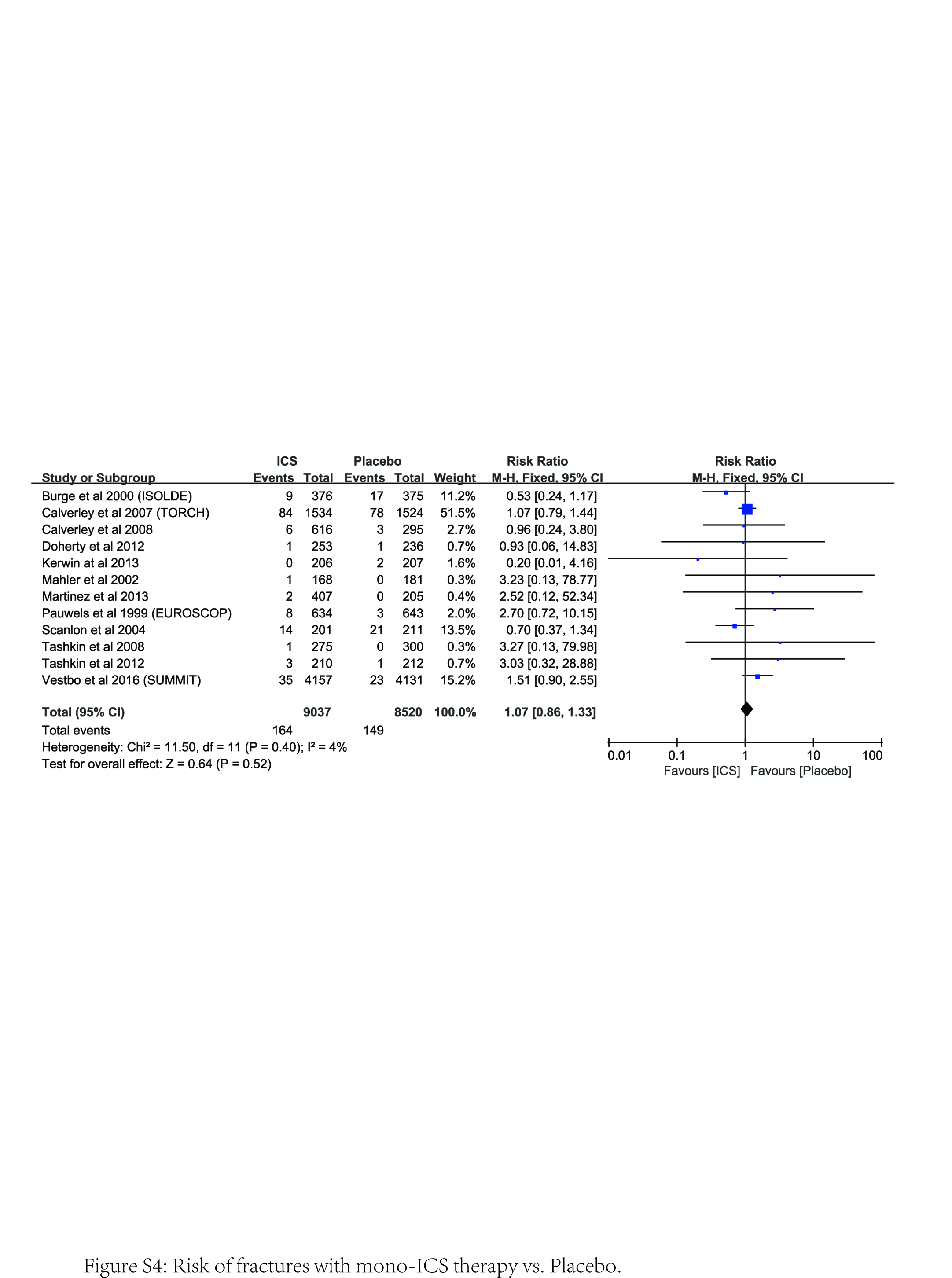

Supplement: Supplementary file 7 — Additional file 7: Figure S4. Riskof fractures with mono-ICS therapy vs. Placebo. [file 12890_2023_2602_MOESM7_ESM.tif]

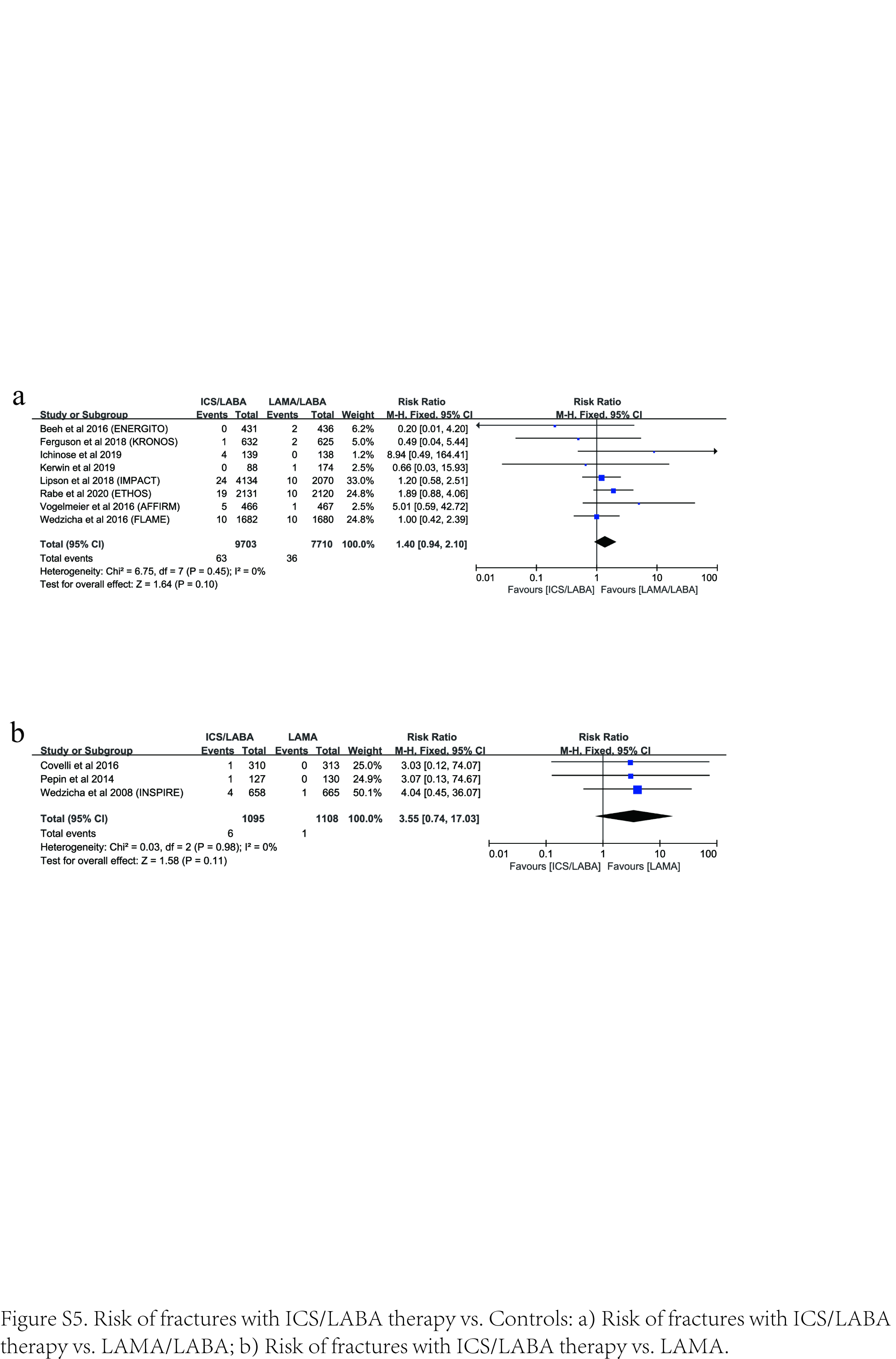

Supplement: Supplementary file 8 — Additional file 8: Figure S5. Risk of fractures with ICS/LABA therapy vs. Controls: a) Risk of fractures with ICS/LABA therapy vs. LAMA/LABA; b) Risk of fractures with ICS/LABA therapy vs. LAMA. [file 12890_2023_2602_MOESM8_ESM.tif]

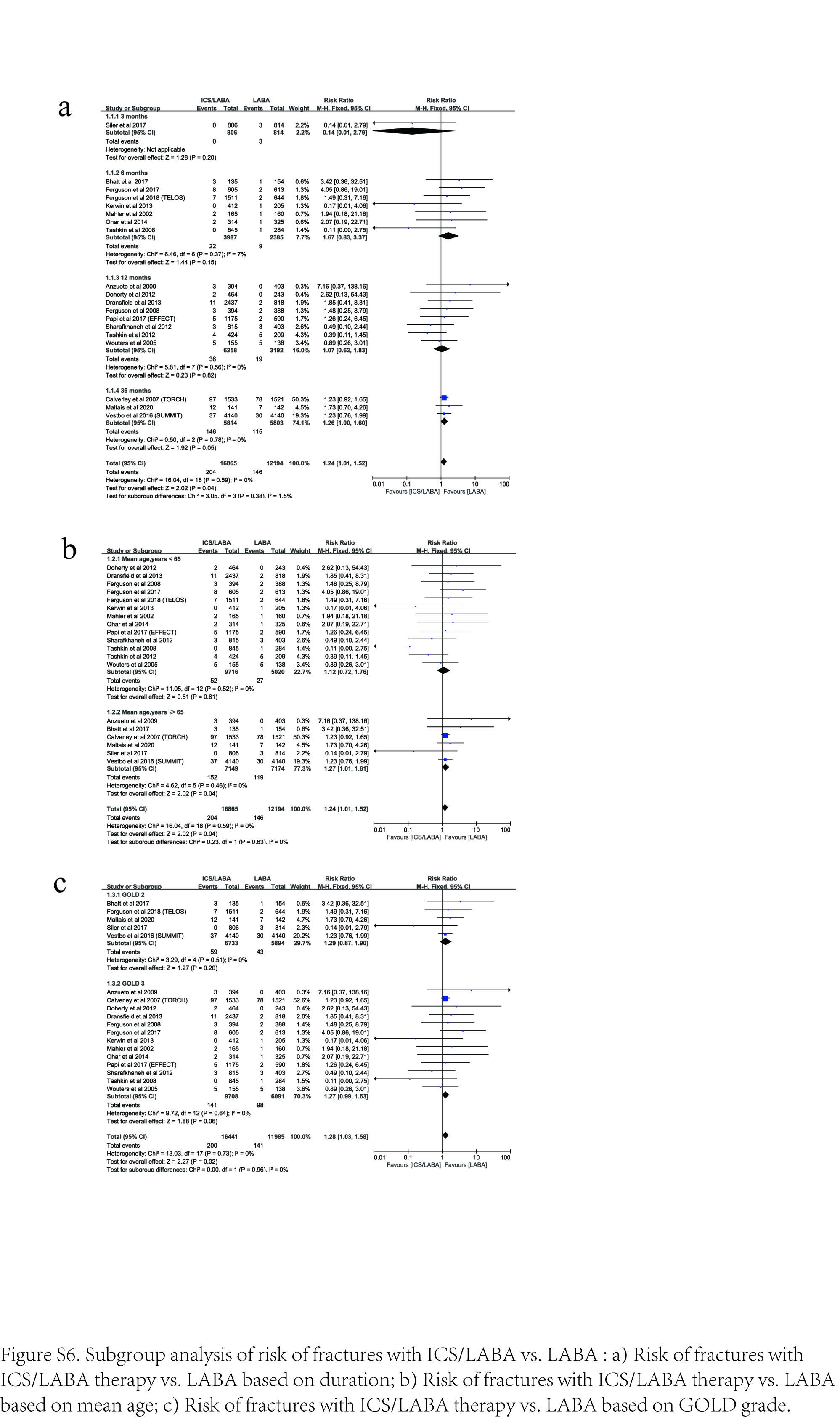

Supplement: Supplementary file 9 — Additional file 9: Figure S6. Subgroup analysis of risk of fractures with ICS/LABA vs. LABA: a) Risk of fractures with ICS/LABA therapy vs. LABA based on duration; b) Risk of fractures with ICS/LABA therapy vs. LABA based on mean age; c) Risk of fractures with ICS/LABA therapy vs. LABA based on GOLD grade. [file 12890_2023_2602_MOESM9_ESM.tif]

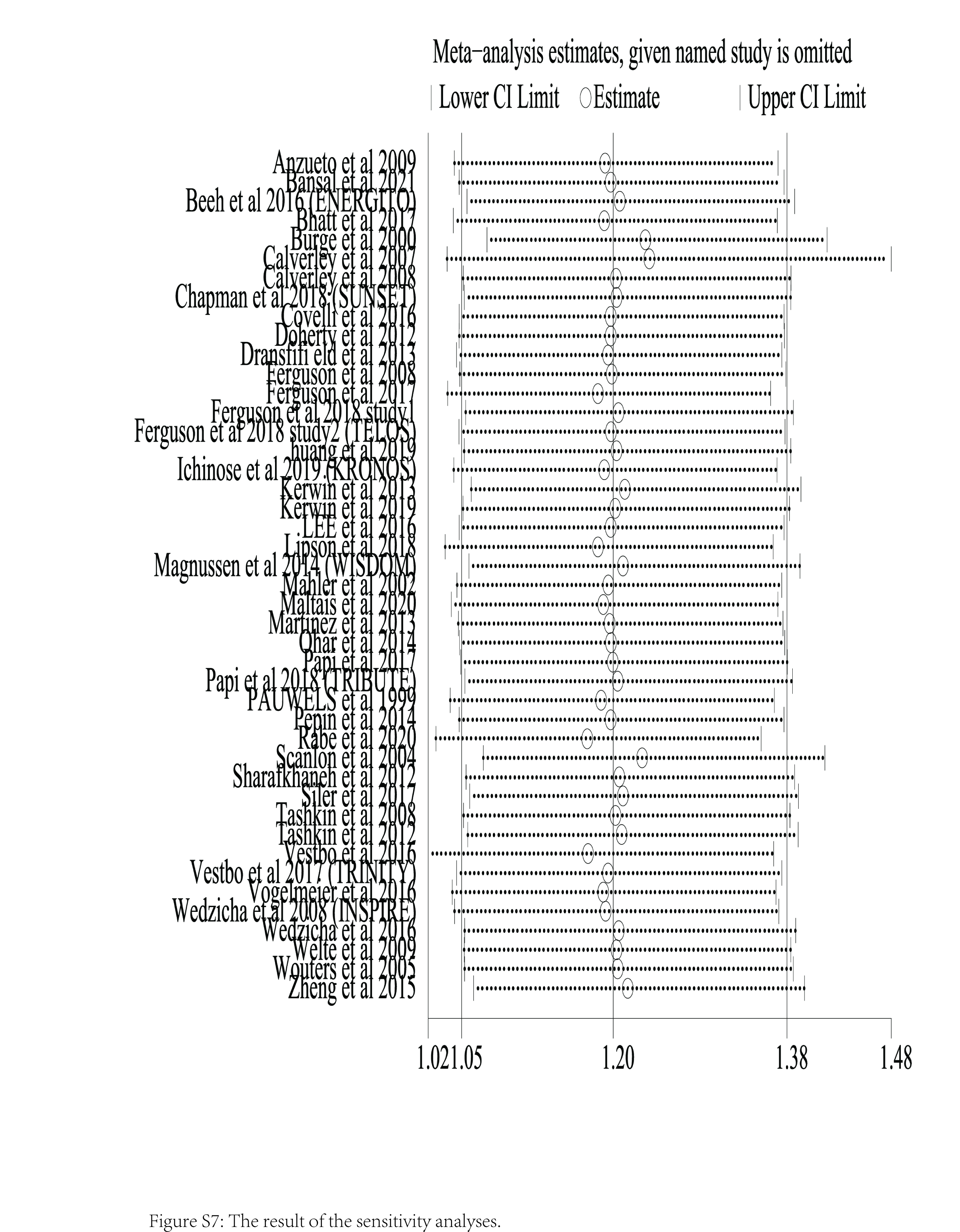

Supplement: Supplementary file 10 — Additional file 10: Figure S7. The result of the sensitivity analyses. [file 12890_2023_2602_MOESM10_ESM.tif]

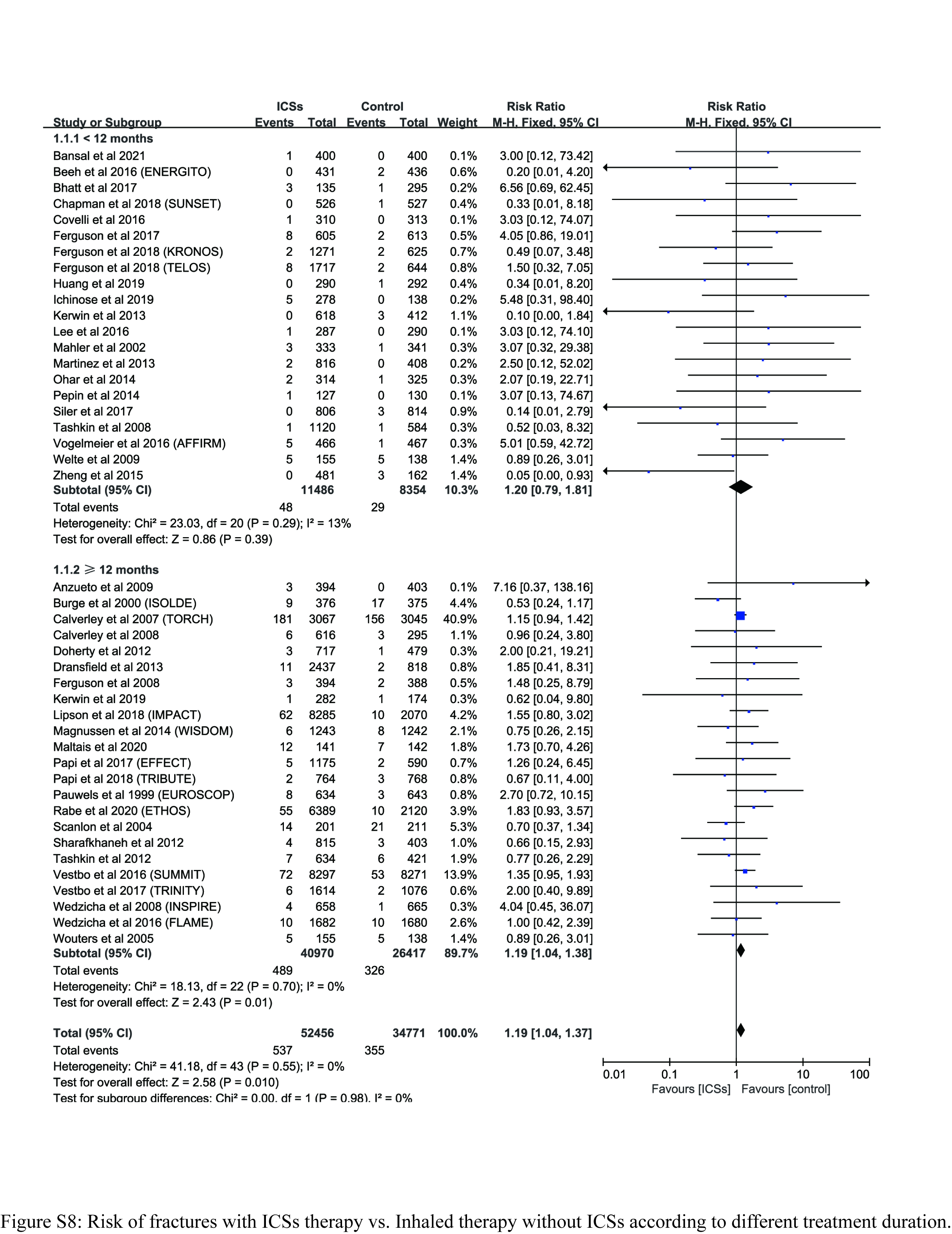

Supplement: Supplementary file 11 — Additional file 11: Figure S8. Risk of fractures with ICSs therapy vs. Inhaled therapy without ICSs according to different treatment duration. [file 12890_2023_2602_MOESM11_ESM.tif]

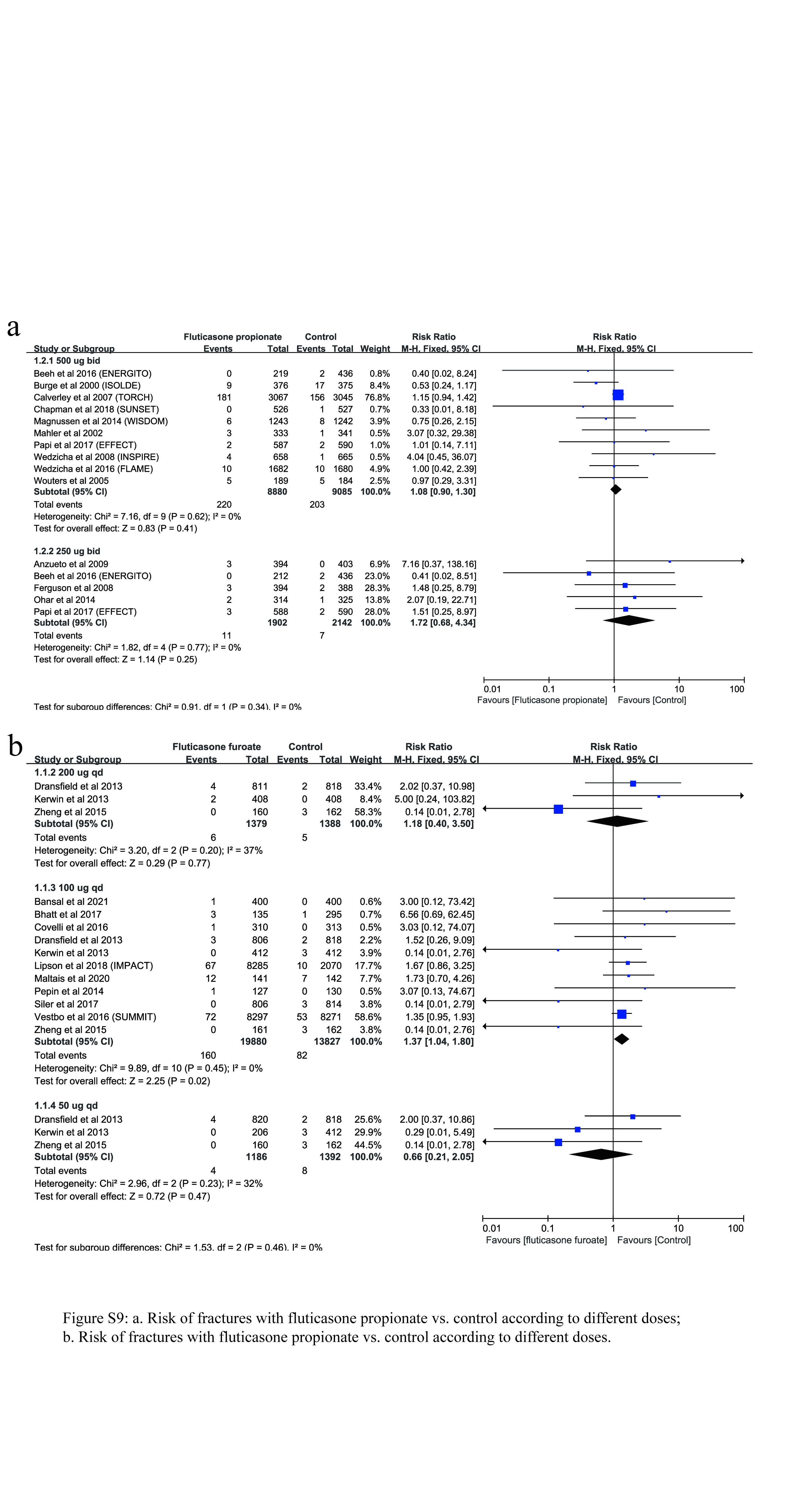

Supplement: Supplementary file 12 — Additional file 12: Figure S9. a. Risk of fractures with fluticasone propionate vs. control according to different doses; b. Risk of fractures with fluticasone propionate vs. control according to different doses. [file 12890_2023_2602_MOESM12_ESM.tif]

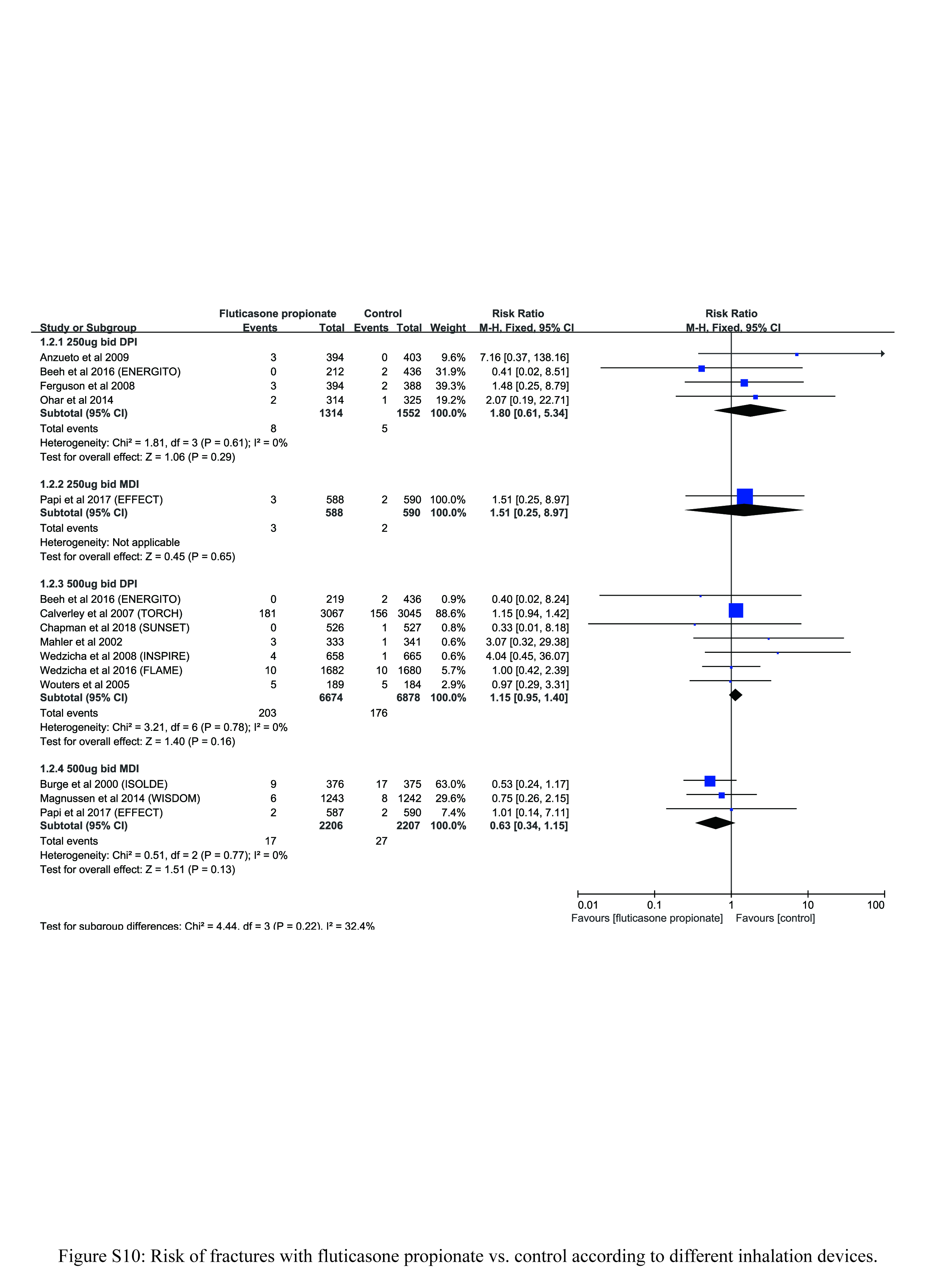

Supplement: Supplementary file 13 — Additional file 13: Figure S10. Risk of fractures with fluticasone propionate vs. control according to different inhalation device. [file 12890_2023_2602_MOESM13_ESM.tif]
